# Use of Patient-Reported Outcome Measures to Assess Outpatient Postpartum Recovery: A Systematic Review

**DOI:** 10.1001/jamanetworkopen.2021.11600

**Published:** 2021-05-27

**Authors:** Pervez Sultan, Nadir Sharawi, Lindsay Blake, Kazuo Ando, Ellile Sultan, Nima Aghaeepour, Brendan Carvalho, Nishant Sadana

**Affiliations:** 1 Department of Anesthesiology, Perioperative and Pain Medicine, Stanford University School of Medicine, Stanford, California; 2 Department of Anesthesiology, University of Arkansas for Medical Sciences, Little Rock; 3 Clinical Services,University of Arkansas for Medical Sciences, Little Rock; 4 University of Illinois, Chicago

## Abstract

**Importance:**

Outpatient postpartum recovery is an underexplored area of obstetrics. There is currently no consensus regarding which patient-reported outcome measure (PROM) clinicians and researchers should use to evaluate postpartum recovery.

**Objective:**

To evaluate PROMs of outpatient postpartum recovery using Consensus-Based Standards for the Selection of Health Measurement Instruments (COSMIN) guidelines.

**Evidence Review:**

An initial literature search performed in July 2019 identified postpartum recovery PROMs and validation studies. A secondary search in July 2020 identified additional validation studies. Both searches were performed using 4 databases (Web of Science, Embase, PubMed, and CINAHL), with no date limiters. Studies with PROMs evaluating more than 3 proposed outpatient postpartum recovery domains were considered. Studies were included if they assessed any psychometric measurement property of the included PROMs in the outpatient postpartum setting. The PROMs were assessed for the following 8 psychometric measurement properties, as defined by COSMIN: content validity, structural validity, internal consistency, cross-cultural validity and measurement invariance, reliability, measurement error, hypothesis testing, and responsiveness. Psychometric measurement properties were evaluated in each included study using the COSMIN criteria by assessing (1) the quality of the methods (very good, adequate, doubtful, inadequate, or not assessed); (2) overall rating of results (sufficient, insufficient, inconsistent, or indeterminate); (3) level of evidence assessed using the Grading of Recommendations, Assessment, Development and Evaluations assessment tool; and (4) level of recommendation, which included class A (recommended for use; showed adequate content validity with at least low-quality evidence for sufficient internal consistency), class B (not class A or class C), or class C (not recommended).

**Findings:**

In total, 15 PROMs (7 obstetric specific and 8 non–obstetric specific) were identified, evaluating outpatient postpartum recovery in 46 studies involving 19 165 women. The majority of psychometric measurement properties of the included PROMs were graded as having very-low-level or low-level evidence. The best-performing PROMs that received class A recommendations were the Maternal Concerns Questionnaire, the Postpartum Quality of Life tool, and the World Health Organization Quality of Life–BREF. The remainder of the evaluated PROMs had insufficient evidence to make recommendations regarding their use (and received class B recommendations).

**Conclusions and Relevance:**

This review found that the best-performing PROMs currently available to evaluate outpatient postpartum recovery were the Maternal Concerns Questionnaire, the Postpartum Quality of Life tool, and the World Health Organization Quality of Life–BREF; however, these tools all had significant limitations. This study highlights the need to focus future efforts on robustly developing and validating a new PROM that may comprehensively evaluate outpatient postpartum recovery.

## Key Points

**Question:**

Using Consensus-Based Standards for the Selection of Health Measurement Instruments guidelines, what is the best patient-reported outcome measure (PROM) to evaluate outpatient postpartum recovery?

**Findings:**

This systematic review identified 15 PROMs evaluating outpatient postpartum recovery in 46 studies, involving 19 165 patients, with most psychometric measurement properties of included PROMs graded very-low-level or low-level evidence. The best-performing PROMs were the Maternal Concerns Questionnaire, the Postpartum Quality of Life tool, and the World Health Organization Quality of Life-BREF; however, these all have significant limitations.

**Meaning:**

There is a need to robustly develop and validate a PROM that can comprehensively evaluate outpatient postpartum recovery.

## Teaser

This systematic review uses Consensus-Based Standards for the Selection of Health Measurement Instruments guidelines to identify the best available multidomain patient-reported outcome measure tools that assess maternal postpartum recovery beyond delivery hospitalization.

## Introduction

An estimated 353 000 mothers give birth each day globally.^1^ Postpartum obstetric care therefore accounts for a substantial percentage of global health care expenditure. Postpartum recovery is a multidimensional process, which is associated with women’s long-term physical, psychosocial, and emotional health and infants’ physical and social development.^2,3^ Morbidity in the outpatient setting is common following childbirth and frequently persists beyond 6 months post partum.^4,5,6^ Despite this, for most women, the 6-week postpartum appointment remains the only contact with the health care system following childbirth. Although outpatient postpartum recovery domains (aspects or dimensions constituting postpartum recovery) have been proposed (physical function, pain, psychosocial distress, surgical or medical factors, infant health, appearance or cosmetic factors, feeding or breast health, psychosocial support, motherhood experience, sleep, fatigue, cognition, and sexual function),^7^ limited guidance is available to clinicians regarding how to best assess postpartum recovery.

Patient-reported outcome measures (PROMs), structured questionnaires enabling patients to report their health status, are now widely regarded as the gold standard means of assessing recovery following surgery.^8^ Although PROMs have been developed and validated to assess inpatient postpartum recovery,^7,9,10,11,12,13^ to our knowledge, there is currently no consensus regarding which PROM optimally measures the quality of recovery for outpatient postpartum women.^14,15^ Systematic review and psychometric analysis of available PROMs may help inform clinicians and researchers regarding whether a suitable measure exists that is capable of adequately assessing outpatient postpartum recovery. Such a multidomain PROM may be useful for screening and identifying women who are recovering poorly and may benefit from targeted interventions and further physician input. Developing a standardized, multidimensional PROM will address an unmet need, which may help reduce morbidity and improve quality of life in the outpatient postpartum period.

We performed a systematic review of validated PROMs used to assess more than 3 domains of outpatient postpartum recovery, using Consensus-Based Standards for the Selection of Health Measurement Instruments (COSMIN) methods,^16^ to determine the best PROM for use after hospital discharge.

## Methods

This review was registered with PROSPERO (CRD42020180533) and follows the Preferred Reporting Items for Systematic Reviews and Meta-analyses (PRISMA) reporting guidelines and Consensus-Based Standards for the Selection of Health Measurement Instruments (COSMIN) guidelines for performing systematic reviews of PROMs.^16^ Recovery for purposes of this review was classified into 13 domains adapted from previously proposed postpartum recovery domains as already stated. A comprehensive list of symptoms for each recovery domain is provided in eTable 1 in the Supplement.

### Search Strategy

A recently published scoping review reported 88 references, which used 17 PROMs of postpartum recovery, all of which evaluated at least 4 postpartum recovery domains.^7^ These articles and their reference lists were screened to identify validation studies and additional PROMs (eAppendix 1 in the Supplement). After the initial search in July 2019, we performed a secondary search using Web of Science, Embase, PubMed, and CINAHL. This search was performed by a medical librarian on July 11, 2020, to identify all validation studies and additional outpatient PROMs that have been used to evaluate more than 3 domains of postpartum recovery. The search strategy included terms and alternative spellings related to cesarean delivery, operative vaginal delivery, spontaneous vaginal delivery, and postpartum recovery (eAppendix 2 in the Supplement). We also searched ClinicalTrials.gov for postpartum validation studies of identified PROMs.

### Inclusion and Exclusion Criteria

To be considered an appropriate measure of outpatient postpartum recovery, the PROM required question items covering more than 3 of the aforementioned 13 postpartum recovery domains (ie, it is a multidimensional measure of outpatient postpartum recovery). We sought validation studies for outpatient postpartum recovery PROMs. We included studies that assessed 1 or more psychometric measurement properties of the identified PROMs used in the outpatient postpartum setting. Articles were screened to determine whether they included any psychometric assessment of the PROMs. Eight psychometric measurement properties were sought and assessed (as defined by COSMIN criteria^16^): structural validity (model fit of a factor analysis); internal consistency (interrelatedness among PROM items); cross-cultural validity (performance of items on a translated or culturally adapted PROM) or measurement invariance (whether respondents from different groups respond similarly to a particular item); reliability (ability of a PROM to distinguish between patients); measurement error (systemic and random error of an individual patient’s score not associated with true changes in recovery); criterion validity (how PROM scores compare to a gold standard measure); hypothesis testing (degree to which scores are consistent with hypotheses, outcomes, or aims stated in the article Introduction or Methods section [eg, correlation between PROMs or difference in scores between groups]); and responsiveness (ability to detect change over time between 2 postpartum time points).

Studies not using a recovery PROM evaluating more than 3 domains or not performing some form of psychometric analysis were excluded. For example, studies that used a PROM as part of an observational study to report health state, with no psychometric analysis, were excluded. We excluded PROMs reporting aspects of recovery associated with a single domain, such as the physical domain of the 36-item Short Form Health Survey (SF-36). We also excluded studies not published in English and studies evaluating recovery in the inpatient or antepartum settings (≤5 days post partum). We excluded editorials, letters, theses, and abstracts from scientific meetings and questionnaires that were developed to measure patient satisfaction. Finally, we excluded PROMs that consisted of free-text answers rather than predetermined item responses, such as with Likert scales, because these answers could not be easily compared between patient cohorts and because of the limited interpretability of such PROMs.

### Data Extraction

Shortlisted articles were entered into the Rayyan reviewing system online.^17^ A standardized data collection tool was used by 3 authors (P.S., N. Sharawi, and N. Sadana) to extract data from the included studies.

As recommended by the COSMIN checklist, assessment of each PROM was achieved by (1) assessment of each included study for risk of bias associated with each of the 8 psychometric measurement properties, with the quality of individual studies (graded as very good, adequate, doubtful, inadequate, or not assesed) evaluated based on methods used to assess the psychometric measurement property (or properties) of the PROM in each study; (2) psychometric measurement property assessment of the quality of the PROM using the COSMIN checklist, with the PROM graded as sufficient, insufficient, inconsistent, or indeterminate based on results reported in individual studies (ie, how the PROM performed following psychometric measurement property assessment in each study); (3) summarizing pooled results from 1 and 2 for each PROM and each of the 8 psychometric measurement properties, to assess the overall PROM quality as sufficient, insufficient, inconsistent, or indeterminate and grading of quality of evidence as high, moderate, low, or very low based on all available studies; and (4) producing summary of findings tables that were then used to make recommendations regarding the selection of the most appropriate PROM to evaluate outpatient postpartum recovery.

Adequate content validity was determined if a PROM evaluated at least 66% of postpartum recovery domains (≥9 of 13 domains) as assessed by study authors. Internal consistency was evaluated using total scores wherever possible. If individual domain scores were assessed, then PROMs for this outcome were graded as sufficient if Cronbach α for most domains was at least 0.7. Similarly, for hypotheses tested in multiple studies, a PROM was graded as “sufficient” if most results were in accordance with the tested hypotheses.

Grading of the level of evidence was based on assessment of risk of bias, inconsistency, imprecision, and indirectness. Studies with response rates of 70% or higher were not downgraded for selection bias as decided a priori by study authors. For studies that failed to report missing data, the numbers approached and the response rates were downgraded accordingly owing to potential for selection bias.

Recommendations were based on each PROM being classified as A, B, or C as follows: (A) PROMs with evidence for sufficient content validity (any level) and at least low-quality evidence for sufficient internal consistency; (B) PROMs categorized not in A or C; and (C) PROMs with high-quality evidence for an insufficient measurement property. PROMs categorized as A were recommended for use, and results obtained with these PROMs may be trusted. PROMs categorized as B had the potential to be recommended for use but required further research to assess their quality. PROMs categorized as C were not recommended for use.

## Results

A flow diagram summarizing the 46 included studies and PROMs evaluated in this review is provided in the Figure.^18,19,20,21,22,23,24,25,26,27,28,29,30,31,32,33,34,35,36,37,38,39,40,41,42,43,44,45,46,47,48,49,50,51,52,53,54,55,56,57,58,59,60,61,62,63^ There were 1277 postpartum studies registered with ClincalTrials.gov, none of which were validation studies for any of the identified PROMs. Three PROMs from the previous scoping review were excluded (1 required free-text responses, and 2 evaluated multiple domains primarily associated with sexual function rather than overall postpartum recovery). Searching of reference lists led to identification of 1 additional PROM (Postpartum Quality of Life [PQOL]). The secondary search specific to the 15 identified PROMs plus a review of the reference lists of included studies identified 9 additional validation studies. The reasons for study exclusion are provided in eTable 2 in the Supplement.

**Figure.**
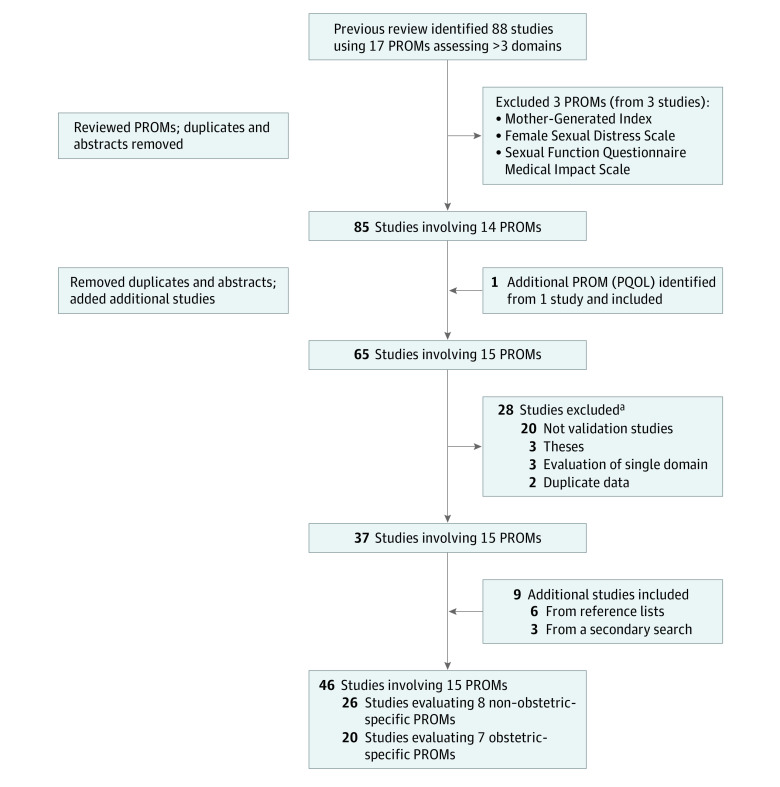
Summary of Search PQOL represents Postpartum Quality of Life; PROMs, patient-reported outcome measures. ^a^See eTable 2 in the Supplement.

### Summary of Included Studies Evaluating Psychometric Measurement Properties

Table 1 summarizes the included studies grouped by the 15 recovery PROMs identified, which underwent psychometric measurement property evaluation in the postpartum period. Further details regarding the 46 studies meeting inclusion criteria for this review, including country of study, language of PROM evaluated, participant numbers, and PROM response rates, are given in eTable 3 in the Supplement.

**Table 1.** Studies Using Each Patient-Reported Outcome Measure^a^

Measure	Obstetric-specific tool	No. of validation studies^b^	No. of patients	No. of studies and study design	Language of instrument (No.)	Study years, range	Delivery mode	Postpartum point assessed
**Obstetric-specific measures**								
Inventory of Functional Status After Childbirth	Yes	7	1066^c^	7 Prospective	English (4); Malay (2); Iranian (1)	1988-2016	Not stated	2 wk to 6 mo
Barkin Index	Yes	6	1139	6 Prospective	English (4); Iranian (2)	2010-2020	Not stated	4 wk to 1 y
Maternal Postpartum QOL	Yes	2	184	2 Prospective (2 studies from 1 data set)	English (2)	2006-2007	All	Weeks 1 and 3
Postpartum symptom checklist	Yes	1	106	1 Prospective	English (1)	2005	All	Day 2 and weekly from weeks 1-6
Maternal Concerns Questionnaire	Yes	2	287	2 Prospective (1 study included data from 5 theses)	English (1)	1995-1998	Not stated	Day 3 to week 8
Rural Postpartum QOL	Yes	1	1375	1 Prospective	Chinese (language not stated) (1)	2012	All	0-3, 4-6, and 7-12 mo
Postpartum QOL	Yes	1	500	1 Prospective	Chinese (language not stated) (1)	2016	All	8 wk
**Non–obstetric-specific measures**								
36-Item Short Form Health Survey	No	9	2512^d^	9 Prospective	English (2); Iran (3); Dutch (3); Spanish (1)	2000-2019	All	Day 0 to 6 y
12-Item Short Form Health Survey	No	7	7496	6 Prospective (1 cluster randomized)	English (4); Taiwanese (1); Malay (1); French (1)	2007-2019	All	Day 1 to 12 y
EQ-5D	No	8	3127	8 Prospective (2 RCTs)	English (4); Dutch (2); Hindi (1); Swedish (1)	2007-2018	All	Day 3 to 12 mo
WHOQOL-BREF	No	1	221	1 Prospective	English (1)	2010	All	Week 6
Nottingham Health Profile	No	1	133	1 Prospective	English (1)	2013	All	Day 1 to 2 wk
WHO Disability Assessment Schedule 2.0	No	1	954	1 Prospective	Ethiopian (language not stated)	2012	Not stated	2 mo
QOL Inventory	No	1	56	1 Prospective RCT	Swedish (1)	2016	All	Weeks 1 and 8
SWED-QUAL	No	1	372	1 Prospective	Swedish (1)	2015	All	5 y

Abbreviations: Barkin Index, Barkin Index of Maternal Functioning; EQ-5D, European quality of life 5-dimension questionnaire; QOL, quality of life; RCT, randomized clinical trial; SWED-QUAL, Swedish Health-Related Quality of Life Survey; WHO, World Health Organization; WHOQOL-BREF, World Health Organization Quality of Life–BREF score.

^a^ Total of 46 validation studies: 1 study evaluated EQ-5D and QOL Inventory^49^ and 2 studies evaluated EQ-5D and 36-item Short Form Health Survey.^33,34^ Hypotheses were considered if measure compared with another measure or evaluated a difference in health state between 2 groups, for example, different delivery mode or following peripartum morbidity (eg, preeclampsia).

^b^ Validation study defined as a study formally assessing and reporting 1 of the 8 COSMIN-defined psychometric measurement properties of patient-reported outcome measures; sum of the number of studies is 49 (3 studies evaluated 2 different PROMs).

^c^ Data from 173 patients used in 2 separate published articles to evaluate Inventory of Functional Status After Childbirth.^30,41^

^d^ Data from 141 patients used in 2 separate published articles to evaluate the 36-item Short Form Health Survey and EQ-5D.^33,34^

Of the 15 PROMs used to evaluate postpartum recovery, 7 were developed for use in the obstetric population,^19,23,25,26,28,61,64^ and 8 were developed in nonobstetric populations. All included studies performed some psychometric measurement property assessment. In total, 19 165 women were evaluated in the 46 included validation studies; 4657 women were evaluated using the 7 obstetric-specific PROMs in 20 studies, and 14 508 women were evaluated using the 8 non–obstetric-specific PROMs in 26 studies.

The SF-36 and the Inventory of Functional Status After Childbirth had the greatest number of validation studies among the non–obstetric-specific PROMs (9 studies) and obstetric-specific PROMs (7 studies). Studies validating the 12-item Short Form Health Survey in the obstetric setting had the highest patient numbers (n = 7496) among all PROMs evaluated. Most of the studies were performed in primarily English-speaking countries and evaluated English versions of PROMs (27 of 46 studies). Twenty-one of the 46 studies evaluated translated versions.

### Risk of Bias for Methods Assessment of the Psychometric Measurement Properties of PROMs

The risk of bias for the assessment of the methods for each psychometric measurement property in each of the included studies is summarized in eTable 4 in the Supplement. The overall quality of the methods for each individual study (risk-of-bias assessment) is provided in eTable 3 in the Supplement. The quality of the methods ranged from inadequate to very good. Most studies used methods in line with COSMIN recommendations to assess the desired psychometric measurement properties of PROMs, and therefore most studies were graded as very good.

### Summary of Psychometric Measurement Properties for Included PROMs

The content validity of each of the 15 included PROMs is summarized in Table 2. The number of postpartum recovery questions included in the PROMs ranged from 6 (European quality of life 5-dimension questionnaire) to 61 (Swedish Health-Related Quality of Life Survey) and covered between 4 and 12 of the predefined 13 postpartum recovery domains. The Maternal Concerns Questionnaire (MCQ) asked questions regarding the greatest number of postpartum recovery domains (12 of 13). The PQOL and the World Health Organization Quality of Life–BREF (WHOQOL-BREF) PROMs also asked questions related to 9 or more of 13 domains (≥66% of domains). Questions regarding the domains of psychosocial distress and psychosocial support were included in most of the PROMs evaluated (13 of 15). Questions regarding the domains of surgical or medical factors, feeding or breast health, appearance or cosmetic, and infant health were included in only a few of the PROMs (3 of 15). Non–obstetric-specific PROMs failed to evaluate surgical or medical factors, motherhood experience, feeding or breast health, and infant health. Cognition and appearance or cosmetic factors were the least frequently covered domains among the obstetric-specific PROMs.

**Table 2.** Content Validity of Included PROMs Used to Evaluate More Than 3 Outpatient Postpartum Recovery Domains

PROM	No. of questions	Likert score	Domains of outpatient postpartum recovery^a^													No. of domains
			Physical	Surgical or medical	Pain	Psychosocial distress	Psychosocial support	Sleep	Motherhood experience	Feeding or breast health	Fatigue	Sexual function	Appearance or cosmetic	Cognition	Infant health	
**Obstetric-specific PROMs**																
IFSAC	36	1 to 4	Yes				Yes	Yes	Yes							4
Barkin Index	20	0 to 6	Yes			Yes	Yes		Yes		Yes					5
MAPPQOL^b^	34	1 to 6			Yes	Yes	Yes			Yes	Yes	Yes			Yes	7
PSC	35	1 to 4	Yes	Yes	Yes	Yes		Yes			Yes			Yes		7
Maternal Concerns Questionnaire^c^	51	1 to 4	Yes	Yes	Yes	Yes	Yes		Yes	Yes	Yes	Yes	Yes	Yes	Yes	12
RPQOL^d^	20	0 to 10		Yes	Yes	Yes	Yes	Yes			Yes	Yes				7
PQOL	40	1 to 5			Yes		Yes	Yes	Yes	Yes	Yes	Yes	Yes		Yes	9
**Non–obstetric PROMs**																
SF-36	36	Variable^e^	Yes		Yes	Yes	Yes				Yes			Yes		6
SF-12	12	Variable^f^	Yes		Yes	Yes	Yes				Yes					5
EQ-5D	5 plus GHVAS	1 to 3; 0 to 100	Yes		Yes	Yes										3
WHOQOL-BREF	26	1 to 5	Yes		Yes	Yes	Yes	Yes			Yes	Yes	Yes	Yes		9
Nottingham HP	45	Yes or no	Yes		Yes	Yes	Yes	Yes			Yes	Yes				7
WHO DAS-II^g^	36 plus 3	1 to 5	Yes			Yes	Yes					Yes		Yes		5
QOL Inventory	16	−6 to 6	Yes			Yes	Yes				Yes			Yes		5
SWED-QUAL	61	1 to 4	Yes		Yes	Yes	Yes	Yes				Yes				6

Abbreviations: Barkin Index, Barkin Index of Maternal Functioning; EQ-5D, European quality of life 5-dimension questionnaire; GHVAS, Global Health Visual Analog Scale; IFSAC, Inventory of Functional Status After Childbirth; MAPPQOL, Maternal Postpartum QOL tool; Nottingham HP, Nottingham Health Profile; PQOL, Postpartum Quality of Life; PROMs, patient-reported outcome measures; PSC, Postpartum Symptoms Checklist; QOL, Quality of Life; RPQOL, Rural Postpartum Quality of Life; SF-12, 12-item Short Form Health Survey; SF-36, 36-item Short Form Health Survey; SWED-QUAL, Swedish Health-Related Quality of Life Survey; WHO DAS-II, World Health Organization Disability Assessment Schedule 2.0; WHOQOL-BREF, World Health Organization Quality of Life–BREF score.

^a^ Yes indicates domain is assesed by the PROM.

^b^ Assessment of satisfaction with different aspects of recovery.

^c^ PROM assesses maternal concerns with different aspects of recovery; also includes an additional (51st) question allowing a free-text answer about additional concerns not listed in the survey.

^d^ No English translated version available but content validity data extracted from factor analysis table presented in a study written in English; only viewpoints of rural women from China included in its design.

^e^ Nine questions 1 to 5; 10 questions 1 to 6; 10 questions 1 to 3; 7 questions 1 to 2.

^f^ Three questions 1 to 5; 3 questions 1 to 6; 4 questions 1 to 2; 2 questions 1 to 3.

^g^ Thirty-six questions 1 to 5 and 3 questions structured as “in the past 30 days, how many days” difficulties were present; SF-36 and SF-12 ask “over past 4 weeks”; WHOQOL-BREF asks about previous 2 weeks; and WHO DAS-II asks about the past 30 days.

The risk of bias, the results, and the overall rating of the results from individual studies are given in eTable 4 in the Supplement. A summary of the main findings of each psychometric measurement property of the 15 evaluated PROMs is also provided.

Structural validity was adequately evaluated in 3 of the included studies. Only the PQOL and Rural Postpartum Quality of Life (RPQOL) PROMs performed well in this psychometric measurement property (root mean square error of approximation, <0.06).

Internal consistency was the most frequently evaluated and assessed psychometric measurement property. Studies reported internal consistency either for total scores or for individual domains. Sufficient internal consistency was shown for all obstetric-specific PROMs and for the SF-36, the WHOQOL-BREF, and the Nottingham Health Profile among the non–obstetric-specific PROMs.

Although several studies used and evaluated psychometric measurement properties of translated versions of PROMs, none of these studies assessed cross-cultural validity using multiple group factor analysis or differential item functioning for group factors, in line with COSMIN recommendations. Similarly, none of the included studies reported measurement invariance using this method.

Sufficient reliability, shown by intraclass correlation coefficients of 0.7 or higher, was reported only in obstetric-specific PROMs with the Inventory of Functional Status After Childbirth, the Barkin Index of Maternal Functioning, and the PQOL. The WHOQOL-BREF showed inadequate reliability with a moderate level of evidence.

Measurement error was not assessed in any of the included validation studies using COSMIN recommended methods. None of the included studies for any of the PROMs defined the minimal clinically important difference in score (the smallest change in score that signifies a clinically significant difference).

Hypothesis testing was performed for all but 2 PROMs (Quality of Life Inventory and MCQ). The majority of PROMs evaluated for this psychometric measurement property compared scores between different modes of delivery at various time points in the postpartum period, ranging from 1 week to 12 years post partum. The other groups compared included women requiring intensive care admission, maternal morbidity (such as preeclampsia), postnatal depression, and urinary incontinence. The Inventory of Functional Status After Childbirth, the Barkin Index of Maternal Functioning, the Maternal Postpartum QOL tool, the PQOL, the SF-36, the European quality of life 5-dimension questionnaire, the WHOQOL-BREF (moderate level of evidence), and the World Health Organization Disability Assessment Schedule 2.0 were the only PROMs that reported “sufficient” results associated with the majority of hypotheses evaluated.

Responsiveness of PROMs was deemed sufficient with the Inventory of Functional Status After Childbirth and SF-36, which were graded with a low level of evidence. This psychometric measurement property was inconsistently evaluated among the included studies. Several studies reported scores at different postpartum times without formal statistical comparison between groups, thus allowing for only qualitative reporting and evaluation. Those studies were scored as inadequate for methods. None of the PROMs showed sufficient consistency in terms of responsiveness or ability to detect change over time. This is likely attributable to a large range in postpartum time points evaluated between studies and different PROMs. It was a requirement for all domains (ie, individual domain scores, when presented and compared) to demonstrate change between time points to be considered to have sufficient responsiveness.

### Feasibility

All PROMs included in this review, except for the QOL Inventory, are available for use in the research or noncommercial setting without cost. Response rates ranged from 27% to 100% (eTable 3 in the Supplement). Missingness of data from completed PROMs was reported in only 2 of the included studies.^29,46^ Response rates were not reported in 14 of the included studies. Five studies reported response rates but did not include information regarding numbers of women approached to participate in the study. Twenty-one studies reported numbers approached and response rate without reporting missing data from surveys completed (eTable 3 in the Supplement). Response rates were higher than 70% in 15 of the included studies.

### Grading of Level of Evidence

Each psychometric measurement property evaluated for all PROMs and the level of evidence for the overall ratings presented are summarized in eTable 5 in the Supplement. The vast majority of evidence from 46 included studies evaluating 15 included PROMs was graded as very low or low. Level of evidence was frequently downgraded owing to observational study design requiring PROM completion in the outpatient setting, which was invariably associated with attrition bias due to inevitable nonresponse. Many studies also recruited convenience samples, predisposing these studies to selection bias. Level of evidence for study outcomes was also downgraded in the presence of unclear response rates. Inconsistency among studies was addressed by rating the results as inconsistent and downgrading the level of evidence by either 1 or 2 levels depending on the context as assessed by the review team. Downgrading by 1 level for imprecision was considered if the total number of patients was less than 100, or by 2 levels if less than 50 patients, per COSMIN guidelines.

### Recommendation

A summary of findings is provided in Table 3. The MCQ, PQOL, and WHOQOL-BREF showed sufficient content validity (assessment of ≥66%; ≥9 of 13 postpartum recovery domains) with low, high, and moderate levels of evidence of sufficient internal consistency, respectively. These 3 PROMs have limitations reducing the strength of recommendation for their routine use.

**Table 3.** Summary of Findings

PROM	Content validity, No. of domains	Structural validity			Internal consistency			Cross-cultural validity or measurement invariance			Reliability			Measurement error			Hypothesis testing			Responsiveness			Recommendation class^b^
		Methods	Results^a^	LoE	Methods	Results^a^	LoE	Methods	Results^a^	LoE	Methods	Results^a^	LoE	Methods	Results^a^	LoE	Methods	Results^a^	LoE	Methods	Results^a^	LoE	
**Obstetric-specific PROMs**																							
IFSAC	4	Very good	I	Mod	Very good	S	Mod	NA	Indeter	Very low	Very good	S	Low	NA	Indeter	Very low	Very good	S	Low	Very good	S	Low	B
Barkin Index	5	Adequate	Indeter	Very low	Very good	S	Low	Inadequate	Indeter	Very low	Very good	S	Low	NA	Indeter	Very low	Very good	S	Low	NA	Indeter	Very low	B
MAPPQOL	7	Adequate	Indeter	Very low	Very good	S	Low	NA	Incons	Very low	Very good	Incons	Mod	NA	Indeter	Very low	Adequate	S	Very low	Inadequate	Indeter	Low	B
PSC	7	Inadequate	Indeter	Very low	Very good	S	Low	NA	Indeter	Very low	NA	Indeter	Very low	NA	Indeter	Very low	Adequate	Indeter	Low	Adequate	Indeter	Low	B
MCQ	12	Inadequate	Indeter	Very low	Very good	S	Low	NA	Indeter	Very low	NA	Indeter	Very low	NA	Indeter	Very low	NA	Indeter	Very low	NA	Indeter	Very low	A
PQOL	9	Very good	S	High	Very good	S	High	NA	Indeter	Very low	Very good	S	High	NA	Indeter	Very low	Very good	S	Mod	NA	Indeter	Very low	A
RPQOL	7	Very good	S	Mod	Very good	S	Mod	NA	Indeter	Very low	NA	Indeter	Very low	NA	Indeter	Very low	Very good	I	Mod	Inadequate	Indeter	Very low	B
**Non–obstetric-specific PROMs**																							
SF-36	6	NA	Indeter	Very low	Very good	S	Mod	NA	Indeter	Very low	Doubtful	Indeter	Very low	NA	Indeter	Very low	Very good	S	Low	Very good	S	Low	B
SF-12	5	NA	Indeter	Very low	NA	Indeter	Very low	NA	Indeter	Very low	NA	Indeter	Very low	NA	Indeter	Very low	Very good	I	Low	Inadequate	Indeter	Very low	B
EQ-5D	3	NA	Indeter	Very low	NA	Indeter	Very low	NA	Indeter	Very low	NA	Indeter	Very low	NA	Indeter	Very low	Very good	S	Low	Inadequate	Incons	Low	B
WHOQOL-BREF	9	NA	Indeter	Very low	Very good	S	Mod	NA	Indeter	Very low	Very good	I	Mod	NA	Indeter	Very low	Very good	S	Mod	NA	Indeter	Very low	A
Nottingham HP	7	NA	Indeter	Very low	Very good	S	Mod	NA	Indeter	Very low	NA	Indeter	Very low	NA	Indeter	Very low	Very good	I	Mod	Inadequate	Indeter	Very low	B
WHO DAS-II	5	NA	Indeter	Very low	NA	Indeter	Very low	NA	Indeter	Very low	NA	Indeter	Very low	NA	Indeter	Very low	Adequate	S	Low	NA	Indeter	Very low	B
QOL Inventory	5	NA	Indeter	Very low	NA	Indeter	Very low	NA	Indeter	Very low	NA	Indeter	Very low	NA	Indeter	Very low	NA	Indeter	Very low	Very good	I	Low	B
SWED-QUAL	6	NA	Indeter	Very low	NA	Indeter	Very low	NA	Indeter	Very low	NA	Indeter	Very low	NA	Indeter	Very low	Very good	Indeter	Low	NA	Indeter	Very low	B

Abbreviations: Barkin Index, Barkin Index of Maternal Functioning; EQ-5D, European quality of life 5-dimension questionnaire; IFSAC, Inventory of Functional Status After Childbirth; I, insufficient, Incons; inconsistent; Indeter, indeterminate; LoE, level of evidence (using the Grading of Recommendations, Assessment, Development and Evaluations assessment tool); MAPPQOL, Maternal Postpartum QOL tool; MCQ, Maternal Concerns Questionnaire; Mod, moderate; Nottingham HP, Nottingham Health Profile; PQOL, Postpartum Quality of Life; NA, not assessed; PROM, patient-reported outcome measure; PSC, Postpartum Symptoms Checklist; QOL, Quality of Life; RPQOL, Rural Postpartum Quality of Life; SF-12, 12-item Short Form Health Survey; S, sufficient; SF-36, 36-item Short Form Health Survey; SWED-QUAL, Swedish Health-Related Quality of Life Survey; WHO DAS-II, World Health Organization Disability Assessment Schedule 2.0; WHOQOL-BREF, World Health Organization Quality of Life–BREF score.

^a^ For overall ratings, S is equivalent to consensus-based standards for the selection of health measurement instruments (COSMIN) guidance +; I, to COSMIN guidance −; Incons, to COSMIN guidance +/−; and Indeter, to COSMIN guidance ?.

^b^ Class A represents evidence for sufficient content validity (any level) and at least low-quality evidence for sufficient internal consistency (PROMs can be recommended for use); class B, PROMs categorized not in class A or C; class C, high-quality evidence for an insufficient measurement property; PROMs with class B recommendation require further evaluation to assess their quality prior to recommendation for use; PROMs with class C recommendation are not recommended for use.

The MCQ is based on data from 5 non–peer-reviewed theses with the number of questions ranging from 46 to 51 and either very low or low levels of evidence. This PROM is based on input from 19 mothers and 6 nurses. The MCQ fails to evaluate sleep; it has not been used beyond 2 weeks post partum; it has limited data regarding its cross-cultural validity, reliability, measurement error, hypothesis testing, and responsiveness; and it has very low levels of evidence supporting findings of its psychometric measurement properties. The time taken to complete the MCQ is 15 to 20 minutes.^27^ Furthermore, the wording of the response options for the MCQ may be open to misinterpretation. The PQOL was developed and evaluated using robust methods and takes 5 to 10 minutes to complete. However, this PROM was developed in China, was validated in Iran, and has limited data regarding measurement error and responsiveness and inadequate evidence of cross-cultural validity, therefore limiting applicability of this PROM in western populations. It also fails to evaluate 4 postpartum recovery domains, and some questions may be less relevant to western cohorts, including “satisfaction with pollution” and “satisfaction with transportation.” Results for hypothesis testing were inconsistent, and no study assessed PQOL responsiveness. The WHOQOL-BREF is not an obstetric-specific PROM and fails to evaluate surgical factors, motherhood experience, feeding, and infant health, which therefore limits its applicability in the postpartum setting. It also shows insufficient reliability (moderate level of evidence), and although it achieved a class A level of recommendation, this was based on data from a single validation study involving 221 patients in Australia.

## Discussion

The main finding of this systematic review is a lack of adequately validated PROMs for evaluation of postpartum recovery. The best available PROMs for outpatient postpartum recovery are the MCQ, PQOL, and WHOQOL-BREF; however, these PROMs all have significant limitations. None of the 15 PROMs assessed in our review evaluated all 13 outpatient postpartum recovery domains. Therefore, to our knowledge, there are currently no PROMs for outpatient postpartum recovery assessment that can be recommended for widespread use. These findings highlight the need to robustly develop and validate a new PROM that comprehensively evaluates outpatient postpartum recovery.

### Clinical Relevance

Approximately 33% of maternal deaths occur between 1 week and 1 year following childbirth, and impaired postpartum recovery may lead to delays in returning to work.^65,66^ The effects of poor postpartum recovery may be associated with impaired long-term women’s health and infant development.^3,4^ There is, therefore, a clinical need to assess recovery in the postpartum period. The use of PROMs is considered to be the gold standard to measure postoperative recovery, and scores on PROMs have been used to guide value-based hospital reimbursements in certain health care systems following elective surgery.^67^ In 2018, the American College of Obstetricians and Gynecologists recommended routine follow-up for all mothers to be performed within 3 weeks of delivery followed by a comprehensive postpartum visit no later than 12 weeks after birth.^68^ A PROM that performs well in measures of validity, reliability, and responsiveness would be invaluable for obstetricians and may help screen patients prior to these consultations to facilitate the direction of the discussion.

A previous review identified 201 PROMs that have been used to evaluate postpartum recovery.^7^ The heterogeneity evident among the PROMs used in postpartum recovery studies justifies the need to perform systematic reviews that evaluate the psychometric measurement properties of existing PROMs and help guide clinicians and researchers to decide which PROM to use. Our recommendations may direct future research efforts by encouraging the use of the most appropriate PROMs with adequate content validity and internal consistency. The use of optimal PROMs may maximize efficiency of research efforts by optimizing the quality of data that can be pooled from future studies and by facilitating interpretation of findings.

### Research Implications

This systematic review provides recommendations regarding which PROMs to consider using when assessing outpatient postpartum recovery. Future studies are needed to identify the best PROMs for use by clinicians and researchers to assess individual postpartum recovery domains, such as depression, pain, and sleep. Although this study identified the MCQ, PQOL, and WHOQOL-BREF as the best available PROMs to evaluate postpartum recovery, the 12 PROMs that received class B recommendations require further psychometric measurement property evaluation. However, given the described limitations of the PROMs that received class A recommendations in this analysis, future research efforts should focus on developing and validating a comprehensive PROM of outpatient postpartum recovery. This may be achieved, for example, by using published methods endorsed by the Patient-Reported Outcomes Measurement Information System initiative (PROMIS).^69^ This process may help determine which domains should be evaluated and whether specific domains should be weighted according to the level of perceived importance to key stakeholders.

### Limitations

Because no gold standard measure of postpartum recovery currently exists, we were unable to assess the criterion validity of included PROMs. We did not exclude studies based on the amount of time after delivery that PROMs were completed, and we acknowledge that this may have influenced the rating for hypothesis testing because the postpartum time point is likely to determine the ability of a PROM to detect differences between groups. We also acknowledge that internal consistency was reported in various ways among the included studies. Some studies reported statistics related to individual domains, whereas other studies reported Cronbach α related to the total PROM score. For studies reporting individual domains, we considered the overall rating to be sufficient if most of the domains had a Cronbach α of 0.7 or higher. We also deemed hypothesis testing to be sufficient if the majority of results presented were in accordance with the hypotheses tested. We believe that, by adopting this pragmatic approach, we consistently assessed the PROMs. Finally, scoring and ranking the quality of the methods, the interpretation of results, and the grading of evidence is a subjective process. However, all included articles were reviewed by 3 authors, and we believe that discussion among these authors resolved discrepancies and reduced variability in interpretation, thereby minimizing the chance of misclassification.

## Conclusions

This analysis provides a detailed assessment of the psychometric measurement properties of the PROMs that have been used to assess outpatient postpartum recovery. The best-performing PROMs were the MCQ, PQOL, and WHOQOL-BREF; however, all of these tools have significant shortcomings. Therefore, this study highlights the need to robustly develop and validate a new PROM that comprehensively evaluates outpatient postpartum recovery.

## Notes

Supplement. **eAppendix 1.** Literature Search Strategy **eAppendix 2.** Updated Search July 11, 2020, for Publications of Validated Measures Since Previous Search **eTable 1.** Factors Associated With Postpartum Recovery Grouped Into Domains **eTable 2.** Summary of Numbers of Included and Excluded Patient-Reported Outcome Measures in This Review **eTable 3.** Summary of Included Studies **eTable 4.** Risk of Bias Assessment of Methods, Reported Results and Overall Rating of Results From All Included Validation Studies **eTable 5.** Summary of Ratings of Overall Psychometric Measurement Properties From Grouped Studies for Each Measure and Grade Assessment of Level of Evidence eReferences.
