# Supplementary material for: Use of Patient-Reported Outcome Measures to Assess Outpatient Postpartum Recovery: A Systematic Review
Source: JAMA Netw Open. 2021 May 27;4(5):e2111600. doi: 10.1001/jamanetworkopen.2021.11600 (PMC8160591; doi:10.1001/jamanetworkopen.2021.11600)
Supplement: Supplement. — eAppendix 1. Literature Search Strategy eAppendix 2. Updated Search July 11, 2020, for Publications of Validated Measures Since Previous Search eTable 1. Factors Associated With Postpartum Recovery Grouped Into Domains eTable 2. Summary of Numbers of Included and Excluded Patient-Reported Outcome Measures in This Review eTable 3. Summary of Included Studies eTable 4. Risk of Bias Assessment of Methods, Reported Results and Overall Rating of Results From All Included Validation Studies eTable 5. Summary of Ratings of Overall Psychometric Measurement Properties From Grouped Studies for Each Measure and Grade Assessment of Level of Evidence eReferences. [file jamanetwopen-e2111600-s001.pdf]

Supplementary Online Content

Sultan P, Sharawi N, Blake L, et al. Use of patient-reported outcome measures to assess outpatient postpartum recovery: a systematic review. *JAMA Netw Open*. 2021;4(5):e2111600. doi:10.1001/jamanetworkopen.2021.11600

eAppendix 1. Literature Search Strategy
eAppendix 2. Updated Search July 11, 2020, for Publications of Validated Measures Since Previous Search
eTable 1. Factors Associated With Postpartum Recovery Grouped Into Domains
eTable 2. Summary of Numbers of Included and Excluded Patient-Reported Outcome Measures in This Review
eTable 3. Summary of Included Studies
eTable 4. Risk of Bias Assessment of Methods, Reported Results and Overall Rating of Results From All Included Validation Studies
eTable 5. Summary of Ratings of Overall Psychometric Measurement Properties From Grouped Studies for Each Measure and Grade Assessment of Level of Evidence
eReferences.

This supplementary material has been provided by the authors to give readers additional information about their work.

eAppendix 1. Literature Search Strategy

PubMed – July 1, 2019 – 6,082 results

("Delivery, Obstetric"[Mesh] OR "Labor, Induced"[Mesh] OR "Extraction, Obstetrical"[Mesh] OR “instrumental delivery” OR “vaginal delivery” OR “vaginal birth” OR “vacuum delivery” OR “vacuum assisted delivery” OR “forceps assisted delivery” OR childbirth) AND ("Postpartum Period"[Mesh] OR "Postnatal Care"[Mesh] OR intrapartum OR postpartum OR postnatal OR "Postoperative Complications"[Mesh] OR "Postoperative Care"[Mesh] OR "Postoperative Period"[Mesh] OR “postoperative” OR “post-operative” or “postsurgery” OR “post surgical” OR “postop” OR recovery OR "Recovery Room"[Mesh] OR "recovery room" OR “post-anesthesia” OR “post-anaesthesia” OR “pacu” OR ((post-anesthesia OR post-anaesthesia) AND acute care unit)) NOT ("Prenatal Care"[Mesh] OR "Prenatal Diagnosis"[Mesh] OR "Prenatal Education"[Mesh] OR prenatal OR antenatal)

AND (("Recovery of Function"[Mesh] OR "Activities of Daily Living"[Mesh] OR "Delayed Emergence from Anesthesia"[Mesh] OR "Fatigue"[Mesh] OR"Anesthesia Recovery Period"[Mesh] OR "Emergence Delirium"[Mesh] OR "Acclimatization"[Mesh] OR "Adaptation, Physiological"[Mesh] OR "Maternal Health"[Mesh] OR “maternal health” OR caretaker OR adaptation OR “fatigue” OR “eat” OR “eating” OR “drink” OR “drinking” OR "Postoperative Nausea and Vomiting"[Mesh] OR "Lethargy"[Mesh] OR nausea OR vomiting))
OR ("Female Urogenital Diseases and Pregnancy Complications"[Mesh] OR "Urinary Incontinence"[Mesh] OR "Uterine Prolapse"[Mesh] OR "Dysmenorrhea"[Mesh] OR "Fecal Incontinence"[Mesh] OR "Gastrointestinal Tract"[Mesh] OR “colorectal” OR “colon” OR rectum OR anus OR urogenital OR urological OR gynecological OR “urinary incontinence” OR “dysmenorrhea” OR “uterine prolapse” OR “fecal incontinence”)
OR ("Pain Management"[Mesh] OR "Pain, Postoperative"[Mesh] OR “pain” OR "Myalgia"[Mesh] OR myalgia OR "Pelvic Pain"[Mesh] OR "Pain"[Mesh] OR “pelvic pain” OR "Headache"[Mesh] OR headache OR "Back Pain"[Mesh] OR “back pain”)
OR (("Mental Health Recovery"[Mesh] OR "Anxiety"[Mesh] OR "Stress Disorders, Post-Traumatic"[Mesh] OR "Depression, Postpartum"[Mesh] OR "Psychotic Disorders"[Mesh] OR "Mood Disorders"[Mesh] OR "Suicidal Ideation"[Mesh] OR "Self-Injurious Behavior"[Mesh] OR "Mental Fatigue"[Mesh] OR "Emotional Adjustment"[Mesh] OR "Pleasure"[Mesh] OR "Happiness"[Mesh] OR "Motivation"[Mesh] OR "Guilt"[Mesh] OR "Social Desirability"[Mesh] OR "Hope"[Mesh] OR "Stress, Psychological"[Mesh] OR "Psychomotor Performance"[Mesh] OR "Appetite"[Mesh] OR "Personal Satisfaction"[Mesh] OR "Adaptation, Psychological"[Mesh] OR "Perception"[Mesh] OR "Bipolar Disorder"[Mesh] OR "Phobic Disorders"[Mesh] OR "Adjustment Disorders"[Mesh] OR "Dissociative Disorders"[Mesh] OR "Dissociative Identity Disorder"[Mesh] OR "Delirium"[Mesh] OR "Neurocognitive Disorders"[Mesh] OR "Cognitive Dysfunction"[Mesh] OR "Behavior"[Mesh] OR "Conduct Disorder"[Mesh] OR "Disruptive, Impulse Control, and Conduct Disorders"[Mesh] OR "Restless Legs Syndrome"[Mesh] OR "Substance-Related Disorders"[Mesh] OR "Personality"[Mesh] OR "Amnesia"[Mesh] OR "Depersonalization"[Mesh] OR "Factitious Disorders"[Mesh] OR "Somatoform Disorders"[Mesh] OR "Conversion Disorder"[Mesh] OR "psychological recovery" OR "mental health recovery" OR “mental fatigue” OR "mental health" OR “depression” OR “depressing” OR “depressed” OR anxiety OR anxious OR perpetual psychosis OR “baby blues” OR “mood disorder” OR “self-harm” OR “suicidal ideation” OR “post-traumatic stress disorder” OR “ptsd” OR psychosis OR fatigue OR “emotional” OR (emotion AND control) OR “enjoy” OR

© 2021 Sultan P et al. *JAMA Network Open*.

enjoyable OR enjoyment OR motivation OR guilt OR wellbeing OR “worth” OR worthless OR worthlessness OR worthy OR “hope” OR hopeful OR hopeless OR hopelessness OR psychomotor OR “self-control” OR appetite OR coping OR perception OR “mania” OR “phobia” OR adjustment OR “reactive” OR dissociative OR “illness anxiety” OR conversion OR factitious OR depersonalization OR amnesia OR somatic OR substance abuse OR “restless leg” OR neurocognitive OR delirium))
OR ("Social Support"[Mesh] OR "Psychosocial Support Systems"[Mesh] OR “social support” OR "Social Adjustment"[Mesh] OR "Social Isolation"[Mesh] OR "Interpersonal Relations"[Mesh] OR “social isolation” OR "Social Adjustment"[Mesh] OR "Social Participation"[Mesh] OR "Social Behavior"[Mesh])
OR ("Sleep"[Mesh] OR “sleep” OR “sleeping” OR “awake” OR insomnia OR narcolepsy OR "Narcolepsy"[Mesh] OR "Sleep Initiation and Maintenance Disorders"[Mesh] OR "Sleep Latency"[Mesh] OR “sleep latency” OR "Sleep Wake Disorders"[Mesh])
OR ("Arousal"[Mesh] OR "Coitus"[Mesh] OR "Sexual Health"[Mesh] OR "Reproductive Health"[Mesh] OR Coitus OR intercourse OR “sexual health” OR "Sexual Dysfunctions, Psychological"[Mesh] OR "Orgasm"[Mesh] OR orgasm OR "Dyspareunia"[Mesh] OR dyspareunia)
OR ("Breast Feeding"[Mesh] OR "Object Attachment"[Mesh] OR "Infant Care"[Mesh] OR “breast feeding” OR bonding OR “infant care”)
AND ((instrumentation[sh] OR methods[sh] OR "Validation Studies"[pt] OR "Comparative Study"[pt] OR "psychometrics"[MeSH] OR psychometr*[tiab] OR clinimetr*[tw] OR clinometr*[tw] OR "outcome assessment (health care)"[MeSH] OR "outcome assessment"[tiab] OR "outcome measure*"[tw] OR "observer variation"[MeSH] OR "observer variation"[tiab] OR "Health Status Indicators"[Mesh] OR "reproducibility of results"[MeSH] OR reproducib*[tiab] OR "discriminant analysis"[MeSH] OR reliab*[tiab] OR unreliab*[tiab] OR valid*[tiab] OR "coefficient of variation"[tiab] OR coefficient[tiab] OR homogeneity[tiab] OR homogeneous[tiab] OR "internal consistency"[tiab] OR (cronbach*[tiab] AND (alpha[tiab] OR alphas[tiab])) OR (item[tiab] AND (correlation*[tiab] OR selection*[tiab] OR reduction*[tiab])) OR agreement[tw] OR precision[tw] OR imprecision[tw] OR "precise values"[tw] OR test-retest[tiab] OR (test[tiab] AND retest[tiab]) OR (reliab*[tiab] AND (test[tiab] OR retest[tiab])) OR stability[tiab] OR interrater[tiab] OR inter-rater[tiab] OR intrarater[tiab] OR intra-rater[tiab] OR intertester[tiab] OR inter-tester[tiab] OR intratester[tiab] OR intra-tester[tiab] OR interobserver[tiab] OR inter-observer[tiab] OR intraobserver[tiab] OR intra-observer[tiab] OR intertechnician[tiab] OR inter-technician[tiab] OR intratechnician[tiab] OR intra-technician[tiab] OR interexaminer[tiab] OR inter-examiner[tiab] OR intraexaminer[tiab] OR intra-examiner[tiab] OR interassay[tiab] OR inter-assay[tiab] OR intraassay[tiab] OR intra-assay[tiab] OR interindividual[tiab] OR inter-individual[tiab] OR intraindividual[tiab] OR intra-individual[tiab] OR interparticipant[tiab] OR inter-participant[tiab] OR intraparticipant[tiab] OR intra-participant[tiab] OR kappa[tiab] OR kappa's[tiab] OR kappas[tiab] OR repeatab*[tw] OR ((replicab*[tw] OR repeated[tw]) AND (measure[tw] OR measures[tw] OR findings[tw] OR result[tw] OR results[tw] OR test[tw] OR tests[tw])) OR generaliza*[tiab] OR generalisa*[tiab] OR concordance[tiab] OR (intraclass[tiab] AND correlation*[tiab]) OR discriminative[tiab] OR "known group"[tiab] OR "factor analysis"[tiab] OR "factor analyses"[tiab] OR "factor structure"[tiab] OR "factor structures"[tiab] OR dimension*[tiab] OR subscale*[tiab] OR (multitrait[tiab] AND scaling[tiab] AND (analysis[tiab] OR analyses[tiab])) OR "item discriminant"[tiab] OR "interscale correlation*"[tiab] OR error[tiab] OR errors[tiab] OR

© 2021 Sultan P et al. *JAMA Network Open*.

"individual variability"[tiab])OR "interval variability"[tiab] OR "rate variability"[tiab] OR (variability[tiab] AND (analysis[tiab] OR values[tiab])) OR (uncertainty[tiab] AND (measurement[tiab] OR measuring[tiab])) OR "standard error of measurement"[tiab] OR sensitiv*[tiab] OR responsive*[tiab] OR (limit[tiab] AND detection[tiab]) OR "minimal detectable concentration"[tiab] OR interpretab*[tiab] OR ((minimal[tiab] OR minimally[tiab] OR clinical[tiab] OR clinically[tiab]) AND (important[tiab] OR significant[tiab] OR detectable[tiab]) AND (change[tiab] OR difference[tiab])) OR (small*[tiab] AND (real[tiab] OR detectable[tiab]) AND (change[tiab] OR difference[tiab])) OR "meaningful change"[tiab] OR "ceiling effect"[tiab] OR "floor effect"[tiab] OR "Item response model"[tiab] OR IRT[tiab] OR Rasch[tiab] OR "Differential item functioning"[tiab] OR DIF[tiab] OR "computer adaptive testing"[tiab] OR "item bank"[tiab] OR "cross-cultural equivalence"[tiab])
NOT (("Delphi Technique"[Mesh] OR "Cross-Sectional Studies"[Mesh] OR "addresses"[Publication Type] OR "biography"[Publication Type] OR "case reports"[Publication Type] OR "comment"[Publication Type] OR "directory"[Publication Type] OR "editorial"[Publication Type] OR "festschrift"[Publication Type] OR "interview"[Publication Type] OR "lectures"[Publication Type] OR "legal cases"[Publication Type] OR "legislation"[Publication Type] OR "letter"[Publication Type] OR "news"[Publication Type] OR "newspaper article"[Publication Type] OR "patient education handout"[Publication Type] OR "popular works"[Publication Type] OR "congresses"[Publication Type] OR "consensus development conference"[Publication Type] OR "consensus development conference, nih"[Publication Type] OR "practice guideline"[Publication Type]) NOT ("animals"[MeSH Terms] NOT "humans"[MeSH Terms]))

Filters: English

Web of Science – July 1, 2019 – 2,114 results
("Obstetric Delivery" OR "Induced Labor" OR "Obstetrical Extraction " OR “instrumental delivery” OR “vaginal delivery” OR “vaginal birth” OR “vacuum delivery” OR “vacuum assisted delivery” OR “forceps assisted delivery” OR childbirth) AND ("Postpartum Period" OR "Postnatal Care" OR intrapartum OR postpartum OR postnatal OR "Postoperative Complications" OR "Postoperative Care" OR "Postoperative Period" OR “postoperative” OR “post-operative” or “postsurgery” OR “post surgical” OR “postop” OR recovery OR "recovery room" OR “post-anesthesia” OR “post-anaesthesia” OR “pacu” OR ((post-anesthesia OR post-anaesthesia) AND acute care unit)) NOT ("Prenatal Care" OR "Prenatal Diagnosis" OR "Prenatal Education" OR prenatal OR antenatal)

AND ("Recovery of Function" OR "Activities of Daily Living" OR "Delayed Emergence from Anesthesia" OR "Fatigue" OR "Anesthesia Recovery Period" OR "Emergence Delirium" OR "Acclimatization" OR "Adaptation, Physiological" OR "Maternal Health" OR caretaker OR adaptation OR “fatigue” OR “eat” OR “eating” OR “drink” OR “drinking” OR "Postoperative Nausea and Vomiting" OR "Lethargy" OR nausea OR vomiting) OR ("Female Urogenital Diseases and Pregnancy Complications" OR "Urinary Incontinence" OR "Uterine Prolapse" OR "Dysmenorrhea" OR "Fecal Incontinence" OR "Gastrointestinal Tract" OR “colorectal” OR “colon” OR rectum OR anus OR urogenital OR urological OR gynecological OR “urinary incontinence” OR “dysmenorrhea” OR “uterine prolapse” OR “fecal incontinence”) OR ("Pain Management" OR "Pain, Postoperative" OR “pain” OR "Myalgia" OR “pelvic pain” OR "Headache" OR “back pain”) OR ("Mental Health Recovery" OR "Anxiety" OR " Post-

© 2021 Sultan P et al. *JAMA Network Open*.

Traumatic Stress Disorders" OR "Postpartum Depression" OR "Psychotic Disorders" OR "Mood Disorders" OR "Suicidal Ideation" OR "Self-Injurious Behavior" OR "Mental Fatigue" OR "Emotional Adjustment" OR "Pleasure" OR "Happiness" OR "Motivation" OR "Guilt" OR "Social Desirability" OR "Stress" OR "Psychomotor Performance" OR "Appetite" OR "Personal Satisfaction" OR " Psychological Adaptation" OR "Perception" OR "Bipolar Disorder" OR "Phobic Disorders" OR "Adjustment Disorders" OR "Dissociative Disorders" OR "Dissociative Identity Disorder" OR "Delirium" OR "Neurocognitive Disorders" OR "Cognitive Dysfunction" OR "Behavior" OR "Conduct Disorder" OR "Disruptive, Impulse Control, and Conduct Disorders" OR "Restless Legs Syndrome" OR "Substance-Related Disorders" OR "Personality" OR "Amnesia" OR "Depersonalization" OR "Factitious Disorders" OR "Somatoform Disorders" OR "Conversion Disorder" OR "psychological recovery" OR "mental health" OR “depression” OR “depressing” OR “depressed” OR anxiety OR anxious OR perpetual psychosis OR “baby blues” OR “mood disorder” OR “self-harm” OR “ptsd” OR psychosis OR fatigue OR “emotional” OR (emotion AND control) OR “enjoy” OR enjoyable OR enjoyment OR wellbeing OR “worth” OR worthless OR worthlessness OR worthy OR “hope” OR hopeful OR hopeless OR hopelessness OR psychomotor OR “self-control” OR coping OR perception OR “mania” OR “phobia” OR adjustment OR “reactive” OR dissociative OR “illness anxiety” OR somatic OR neurocognitive OR delirium) OR ("Social Support" OR "Psychosocial Support Systems" OR "Social Adjustment" OR "Social Isolation" OR "Interpersonal Relations" OR “social isolation” OR "Social Adjustment" OR "Social Participation" OR "Social Behavior") OR ("Sleep" OR “sleeping” OR “awake” OR insomnia OR narcolepsy OR "Sleep Initiation and Maintenance Disorders" OR “sleep latency” OR "Sleep Wake Disorders") OR ("Arousal" OR "Coitus" OR "Sexual Health" OR "Reproductive Health" OR Coitus OR intercourse OR "Sexual Dysfunctions" OR orgasm OR dyspareunia) OR ("Breast Feeding" OR "Object Attachment" OR "Infant Care" OR bonding)
AND (((instrumentation OR methods OR "Validation Studies" OR "Comparative Study" OR "psychometrics" OR psychometr* OR clinimetr* OR clinometr* OR "health care outcome assessment" OR "outcome assessment" OR "outcome measure*" OR "observer variation" OR "observer variation" OR "Health Status Indicators" OR "reproducibility of results" OR reproducib* OR "discriminant analysis" OR reliab* OR unreliab* OR valid* OR "coefficient of variation" OR coefficient OR homogeneity OR homogeneous OR "internal consistency" OR (cronbach* AND (alpha OR alphas)) OR (item AND (correlation* OR selection* OR reduction*)) OR agreement OR precision OR imprecision OR "precise values" OR test-retest OR (test AND retest) OR (reliab* AND (test OR retest)) OR stability OR interrater OR inter-rater OR intrarater OR intra-rater OR intertester OR inter-tester OR intratester OR intra-tester OR interobserver OR inter-observer OR intraobserver OR intra-observer OR intertechnician OR inter-technician OR intratechnician OR intra-technician OR interexaminer OR inter-examiner OR intraexaminer OR intra-examiner OR interassay OR inter-assay OR intraassay OR intra-assay OR interindividual OR inter-individual OR intraindividual OR intra-individual OR interparticipant OR inter-participant OR intraparticipant OR intra-participant OR kappa OR kappa's OR kappas OR repeatab* OR ((replicab* OR repeated) AND (measure OR measures OR findings OR result OR results OR test OR tests)) OR generaliza* OR generalisa* OR concordance OR (intraclass AND correlation*) OR discriminative OR "known group" OR "factor analysis" OR "factor analyses" OR "factor structure" OR "factor structures" OR dimension* OR subscale* OR (multitrait AND scaling AND (analysis OR analyses)) OR "item discriminant" OR "interscale correlation*" OR error OR errors OR "individual variability") OR

© 2021 Sultan P et al. *JAMA Network Open*.

"interval variability" OR "rate variability" OR (variability AND (analysis OR values)) OR (uncertainty AND (measurement OR measuring)) OR "standard error of measurement" OR sensitiv* OR responsive* OR (limit AND detection) OR "minimal detectable concentration" OR interpretab* OR ((minimal OR minimally OR clinical OR clinically) AND (important OR significant OR detectable) AND (change OR difference)) OR (small* AND (real OR detectable) AND (change OR difference)) OR "meaningful change" OR "ceiling effect" OR "floor effect" OR "Item response model" OR IRT OR Rasch OR "Differential item functioning" OR DIF OR "computer adaptive testing" OR "item bank" OR "cross-cultural equivalence"))
NOT ((("Delphi Technique" OR "Cross-Sectional Studies" OR "addresses" OR "biography" OR "case reports" OR "comment" OR "directory" OR "editorial" OR "festschrift" OR "interview" OR "lectures" OR "legal cases" OR "legislation" OR "letter" OR "news" OR "newspaper article" OR "patient education handout" OR "popular works" OR "congresses" OR "consensus development conference" OR "nih consensus development conference" OR "practice guideline") NOT ("animals" NOT "humans")))
Filters: English

CINAHL – July 1, 2019 – 1,035 results
(MH "Labor, Induced" OR MH "Vacuum Extraction, Obstetrical" OR MH "Delivery, Obstetric" OR MH "Vaginal Birth" OR "Delivery, Obstetric" OR "Labor, Induced" OR "Extraction, Obstetrical" OR “instrumental delivery” OR “vaginal delivery” OR “vaginal birth” OR “vacuum delivery” OR “vacuum assisted delivery” OR “forceps assisted delivery” OR childbirth) AND (MH "Postnatal Care" OR MH "Postnatal Period" OR MH "Postoperative Period" OR MH "Postoperative Care" OR MH "Post Anesthesia Care" OR "Postpartum Period" OR "Postnatal Care" OR intrapartum OR postpartum OR postnatal OR "Postoperative Complications" OR "Postoperative Care" OR "Postoperative Period" OR “postoperative” OR “post-operative” or “postsurgery” OR “post surgical” OR “postop” OR recovery OR "recovery room" OR “post-anesthesia” OR “post-anaesthesia” OR “pacu” OR ((post-anesthesia OR post-anaesthesia) AND acute care unit)) NOT ("Prenatal Care" OR "Prenatal Diagnosis" OR "Prenatal Education" OR prenatal OR antenatal)

AND ((MH "Recovery") OR (MH "Anesthesia Recovery") OR (MH "Activities of Daily Living") OR (MH "Physical Activity") OR (MH "Delirium") OR (MH "Adaptation, Physiological") OR (MH "Adaptation, Psychological") OR (MH "Acclimatization") OR (MH "Nausea") OR (MH "Nausea and Vomiting") OR (MH "Vomiting") OR (MH "Appetite") OR (MH "Fatigue") OR (MH "Mental Fatigue") OR (MH "Muscle Fatigue") OR (MH "Incontinence") OR (MH "Uterine Prolapse") OR (MH "Uterine Hemorrhage") OR (MH "Rectal Prolapse") OR (MH "Uterine Inversion") OR (MH "Metrorrhagia") OR (MH "Pelvic Organ Prolapse") OR (MH "Uterine Rupture") OR (MH "Pelvic Pain") OR (MH "Abdominal Pain") OR (MH "Postoperative Pain") OR (MH "Back Pain") OR (MH "Pain") OR (MH "Depression") OR (MH "Depression, Postpartum") OR (MH "Bipolar Disorder") OR (MH "Stress") OR (MH "Stress Disorders, Post-Traumatic") OR (MH "Pleasure") OR (MH "Hope") OR (MH "Optimism") OR (MH "Hopelessness") OR (MH "Self Concept") OR (MH "Personality") OR (MH "Amnesia") OR (MH "Breast Feeding") OR (MH "Mental Health") OR (MH "Sexual Health") OR (MH "Sexual Dysfunction, Female") OR (MH "Coitus") OR (MH "Self-Injurious Behavior") OR (MH "Personal Satisfaction") OR (MH "Sexual Satisfaction") OR (MH "Headache") OR (MH "Muscle Pain") OR (MH "Anxiety") OR (MH "Adjustment Disorders") OR (MH "Dyssomnias") OR (MH "Sleep") OR (MH "Sleep-Wake Transition Disorders") OR

© 2021 Sultan P et al. *JAMA Network Open*.

(MH "Parasomnias") OR (MH "Sleep Arousal Disorders") OR (MH "Sleep Disorders") OR (MH "Social Behavior Disorders") OR (MH "Social Anxiety Disorders") OR (MH "Social Adjustment") OR (MH "Social Isolation") OR (MH "Parent-Infant Bonding") OR (MH "Infant Care") OR (MH "Perception") OR (MH "Dissociative Disorders") OR (MH "Suicidal Ideation") OR (MH "Happiness") OR (MH "Motivation") OR (MH "Phobic Disorders") OR (MH "Cognition Disorders") OR (MH "Behavior") OR (MH "Parental Behavior") OR (MH "Emotions") OR "Recovery of Function" OR "Activities of Daily Living" OR "Delayed Emergence from Anesthesia" OR "Fatigue" OR "Anesthesia Recovery Period" OR "Emergence Delirium" OR "Acclimatization" OR "Adaptation, Physiological" OR "Maternal Health" OR caretaker OR adaptation OR “fatigue” OR “eat” OR “eating” OR “drink” OR “drinking” OR "Postoperative Nausea and Vomiting" OR "Lethargy" OR nausea OR vomiting) OR ("Female Urogenital Diseases and Pregnancy Complications" OR "Urinary Incontinence" OR "Uterine Prolapse" OR "Dysmenorrhea" OR "Fecal Incontinence" OR "Gastrointestinal Tract" OR “colorectal” OR “colon” OR rectum OR anus OR urogenital OR urological OR gynecological OR “urinary incontinence” OR “dysmenorrhea” OR “uterine prolapse” OR “fecal incontinence”) OR ("Pain Management" OR "Pain, Postoperative" OR “pain” OR "Myalgia" OR “pelvic pain” OR "Headache" OR “back pain”) OR ("Mental Health Recovery" OR "Anxiety" OR " Post-Traumatic Stress Disorders" OR "Postpartum Depression" OR "Psychotic Disorders" OR "Mood Disorders" OR "Suicidal Ideation" OR "Self-Injurious Behavior" OR "Mental Fatigue" OR "Emotional Adjustment" OR "Pleasure" OR "Happiness" OR "Motivation" OR "Guilt" OR "Social Desirability" OR "Stress" OR "Psychomotor Performance" OR "Appetite" OR "Personal Satisfaction" OR " Psychological Adaptation" OR "Perception" OR "Bipolar Disorder" OR "Phobic Disorders" OR "Adjustment Disorders" OR "Dissociative Disorders" OR "Dissociative Identity Disorder" OR "Delirium" OR "Neurocognitive Disorders" OR "Cognitive Dysfunction" OR "Behavior" OR "Conduct Disorder" OR "Disruptive, Impulse Control, and Conduct Disorders" OR "Restless Legs Syndrome" OR "Substance-Related Disorders" OR "Personality" OR "Amnesia" OR "Depersonalization" OR "Factitious Disorders" OR "Somatoform Disorders" OR "Conversion Disorder" OR "psychological recovery" OR "mental health" OR “depression” OR “depressing” OR “depressed” OR anxiety OR anxious OR perpetual psychosis OR “baby blues” OR “mood disorder” OR “self-harm” OR “ptsd” OR psychosis OR fatigue OR “emotional” OR (emotion AND control) OR “enjoy” OR enjoyable OR enjoyment OR wellbeing OR “worth” OR worthless OR worthlessness OR worthy OR “hope” OR hopeful OR hopeless OR hopelessness OR psychomotor OR “self-control” OR coping OR perception OR “mania” OR “phobia” OR adjustment OR “reactive” OR dissociative OR “illness anxiety” OR somatic OR neurocognitive OR delirium) OR ("Social Support" OR "Psychosocial Support Systems" OR "Social Adjustment" OR "Social Isolation" OR "Interpersonal Relations" OR “social isolation” OR "Social Adjustment" OR "Social Participation" OR "Social Behavior") OR ("Sleep" OR “sleeping” OR “awake” OR insomnia OR narcolepsy OR "Sleep Initiation and Maintenance Disorders" OR “sleep latency” OR "Sleep Wake Disorders") OR ("Arousal" OR "Coitus" OR "Sexual Health" OR "Reproductive Health" OR Coitus OR intercourse OR "Sexual Dysfunctions" OR orgasm OR dyspareunia) OR ("Breast Feeding" OR "Object Attachment" OR "Infant Care" OR bonding)
AND (((instrumentation OR methods OR "Validation Studies" OR "Comparative Study" OR "psychometrics" OR psychometr* OR clinimetr* OR clinometr* OR "health care outcome assessment" OR "outcome assessment" OR "outcome measure*" OR "observer variation" OR "observer variation" OR "Health Status Indicators" OR "reproducibility of results" OR

© 2021 Sultan P et al. *JAMA Network Open*.

reproducib* OR "discriminant analysis" OR reliab* OR unreliab* OR valid* OR "coefficient of variation" OR coefficient OR homogeneity OR homogeneous OR "internal consistency" OR (cronbach* AND (alpha OR alphas)) OR (item AND (correlation* OR selection* OR reduction*)) OR agreement OR precision OR imprecision OR "precise values" OR test-retest OR (test AND retest) OR (reliab* AND (test OR retest)) OR stability OR interrater OR inter-rater OR intrarater OR intra-rater OR intertester OR inter-tester OR intratester OR intra-tester OR interobserver OR inter-observer OR intraobserver OR intra-observer OR intertechnician OR inter-technician OR intratechnician OR intra-technician OR interexaminer OR inter-examiner OR intraexaminer OR intra-examiner OR interassay OR inter-assay OR intraassay OR intra-assay OR interindividual OR inter-individual OR intraindividual OR intra-individual OR interparticipant OR inter-participant OR intraparticipant OR intra-participant OR kappa OR kappa's OR kappas OR repeatab* OR ((replicab* OR repeated) AND (measure OR measures OR findings OR result OR results OR test OR tests)) OR generaliza* OR generalisa* OR concordance OR (intraclass AND correlation*) OR discriminative OR "known group" OR "factor analysis" OR "factor analyses" OR "factor structure" OR "factor structures" OR dimension* OR subscale* OR (multitrait AND scaling AND (analysis OR analyses)) OR "item discriminant" OR "interscale correlation*" OR error OR errors OR "individual variability") OR "interval variability" OR "rate variability" OR (variability AND (analysis OR values)) OR (uncertainty AND (measurement OR measuring)) OR "standard error of measurement" OR sensitiv* OR responsive* OR (limit AND detection) OR "minimal detectable concentration" OR interpretab* OR ((minimal OR minimally OR clinical OR clinically) AND (important OR significant OR detectable) AND (change OR difference)) OR (small* AND (real OR detectable) AND (change OR difference)) OR "meaningful change" OR "ceiling effect" OR "floor effect" OR "Item response model" OR IRT OR Rasch OR "Differential item functioning" OR DIF OR "computer adaptive testing" OR "item bank" OR "cross-cultural equivalence"))
NOT ((("Delphi Technique" OR "Cross-Sectional Studies" OR "addresses" OR "biography" OR "case reports" OR "comment" OR "directory" OR "editorial" OR "festschrift" OR "interview" OR "lectures" OR "legal cases" OR "legislation" OR "letter" OR "news" OR "newspaper article" OR "patient education handout" OR "popular works" OR "congresses" OR "consensus development conference" OR "nih consensus development conference" OR "practice guideline") NOT ("animals" NOT "humans")))
Filters: English, Academic Journals and Dissertations

EMBASE – July 1, 2019 – 981 results
('delivery, obstetric'/de OR 'labor, induced'/de OR 'extraction, obstetrical'/de OR 'instrumental delivery'/de OR 'vaginal delivery'/de OR 'vaginal birth'/de OR 'vacuum delivery'/de OR 'vacuum assisted delivery'/de OR 'forceps assisted delivery' OR 'childbirth'/de) AND ('postpartum period'/de OR 'postnatal care'/de OR intrapartum OR 'postpartum'/de OR postnatal OR 'postoperative complications'/de OR 'postoperative care'/de OR 'postoperative period'/de OR 'postoperative' OR 'post-operative' OR 'postsurgery' OR 'post surgical' OR 'postop' OR 'recovery'/de OR 'recovery room'/de OR 'post-anesthesia' OR 'post-anaesthesia' OR 'pacu' OR (('post anesthesia' OR 'post anaesthesia') AND acute AND 'care'/de AND 'unit'/de))

AND 'recovery of function'/de OR 'activities of daily living'/de OR 'delayed emergence from anesthesia'/de OR 'anesthesia recovery period'/de OR 'emergence delirium'/de OR 'acclimatization'/de OR 'adaptation, physiological'/de OR 'maternal health'/de OR caretaker OR 'adaptation'/de OR 'eat' OR 'eating'/de OR 'drink' OR 'drinking'/de OR 'postoperative nausea and

© 2021 Sultan P et al. *JAMA Network Open*.

vomiting'/de OR 'lethargy'/de OR 'nausea'/de OR 'vomiting'/de OR 'female urogenital diseases and pregnancy complications'/de OR 'gastrointestinal tract'/de OR 'colorectal' OR 'colon'/de OR 'rectum'/de OR 'anus'/de OR urogenital OR urological OR gynecological OR 'urinary incontinence'/de OR 'dysmenorrhea'/de OR 'uterine prolapse'/de OR 'fecal incontinence'/de OR 'pain management'/de OR 'pain, postoperative'/de OR 'pain'/de OR 'myalgia'/de OR 'pelvic pain'/de OR 'headache'/de OR 'back pain'/de OR (('mental health recovery'/de OR 'post-traumatic stress disorders' OR 'postpartum depression'/de OR 'psychotic disorders'/de OR 'mood disorders'/de OR 'suicidal ideation'/de OR 'self-injurious behavior'/de OR 'mental fatigue'/de OR 'emotional adjustment'/de OR 'pleasure'/de OR 'happiness'/de OR 'motivation'/de OR 'guilt'/de OR 'social desirability'/de OR 'stress'/de OR 'psychomotor performance'/de OR 'appetite'/de OR 'personal satisfaction'/de OR 'psychological adaptation'/de OR 'perception'/de OR 'bipolar disorder'/de OR 'phobic disorders'/de OR 'adjustment disorders'/de OR 'dissociative disorders'/de OR 'dissociative identity disorder'/de OR 'delirium'/de OR 'neurocognitive disorders'/de OR 'cognitive dysfunction'/de OR 'behavior'/de OR 'conduct disorder'/de OR 'disruptive, impulse control, and conduct disorders'/de OR 'restless legs syndrome'/de OR 'substance-related disorders'/de OR 'personality'/de OR 'amnesia'/de OR 'depersonalization'/de OR 'factitious disorders'/de OR 'somatoform disorders'/de OR 'conversion disorder'/de OR 'psychological recovery' OR 'mental health'/de OR 'depression'/de OR 'depressing' OR 'depressed' OR 'anxiety'/de OR anxious OR perpetual) AND 'psychosis'/de) OR 'baby blues' OR 'mood disorder'/de OR 'self-harm'/de OR 'ptsd'/de OR 'psychosis'/de OR 'fatigue'/de OR 'emotional' OR ('emotion'/de AND 'control'/de) OR 'enjoy' OR enjoyable OR 'enjoyment'/de OR 'wellbeing'/de OR 'worth' OR worthless OR 'worthlessness'/de OR worthy OR 'hope'/de OR hopeful OR hopeless OR 'hopelessness'/de OR psychomotor OR 'self-control'/de OR 'coping'/de OR 'perception'/de OR 'mania'/de OR 'phobia'/de OR 'adjustment'/de OR 'reactive' OR dissociative OR 'illness anxiety' OR somatic OR neurocognitive OR 'delirium'/de OR 'social support'/de OR 'psychosocial support systems'/de OR 'interpersonal relations'/de OR 'social isolation'/de OR 'social adjustment'/de OR 'social participation'/de OR 'social behavior'/de OR 'sleep'/de OR 'sleeping'/de OR 'awake'/de OR 'insomnia'/de OR 'narcolepsy'/de OR 'sleep initiation and maintenance disorders'/de OR 'sleep latency'/de OR 'sleep wake disorders'/de OR 'arousal'/de OR 'sexual health'/de OR 'reproductive health'/de OR 'coitus'/de OR 'intercourse'/de OR 'sexual dysfunctions' OR 'orgasm'/de OR 'dyspareunia'/de OR 'breast feeding'/de OR 'object attachment'/de OR 'infant care'/de OR 'bonding'/de
AND ('instrumentation'/de OR 'methods'/de OR 'validation studies'/de OR 'comparative study'/de OR 'psychometrics'/de OR psychometr* OR clinimetr* OR clinometr* OR 'health care outcome assessment' OR 'outcome assessment'/de OR 'outcome measure*' OR 'observer variation'/de OR 'health status indicators'/de OR 'reproducibility of results'/de OR reproducib* OR 'discriminant analysis'/de OR reliab* OR unreliab* OR valid* OR 'coefficient of variation'/de OR coefficient OR 'homogeneity'/de OR homogeneous OR 'internal consistency'/de OR (cronbach* AND (alpha OR alphas)) OR (item AND (correlation* OR selection* OR reduction*)) OR 'agreement'/de OR 'precision'/de OR imprecision OR 'precise values' OR 'test retest' OR ('test'/de AND retest) OR (reliab* AND ('test'/de OR retest)) OR 'stability'/de OR interrater OR 'inter rater' OR intrarater OR 'intra rater' OR intertester OR 'inter tester' OR intratester OR 'intra tester' OR interobserver OR 'inter observer' OR intraobserver OR 'intra observer' OR intertechnician OR 'inter technician' OR intratechnician OR 'intra technician' OR interexaminer OR 'inter examiner' OR intraexaminer OR 'intra examiner' OR interassay OR 'inter assay' OR intraassay OR 'intra assay' OR interindividual OR 'inter individual' OR intraindividual OR 'intra individual' OR interparticipant

© 2021 Sultan P et al. *JAMA Network Open*.

OR 'inter participant' OR intraparticipant OR 'intra participant' OR kappa OR kappas OR repeatab* OR ((replicab* OR repeated) AND (measure OR measures OR findings OR result OR results OR 'test'/de OR tests)) OR generaliza* OR generalisa* OR 'concordance'/de OR (intraclass AND correlation*) OR discriminative OR 'known group' OR 'factor analysis'/de OR 'factor analyses' OR 'factor structure'/de OR 'factor structures' OR dimension* OR subscale* OR (multitrait AND 'scaling'/de AND ('analysis'/de OR analyses)) OR 'item discriminant' OR 'interscale correlation*' OR 'error'/de OR errors OR 'individual variability' OR 'interval variability' OR 'rate variability' OR ('variability'/de AND ('analysis'/de OR values)) OR ('uncertainty'/de AND ('measurement'/de OR measuring)) OR 'standard error of measurement'/de OR sensitiv* OR responsive* OR (limit AND 'detection'/de) OR 'minimal detectable concentration' OR interpretab* OR ((minimal OR minimally OR 'clinical'/de OR clinically) AND (important OR significant OR detectable) AND ('change'/de OR difference)) OR (small* AND (real OR detectable) AND ('change'/de OR difference)) OR 'meaningful change' OR 'ceiling effect'/de OR 'floor effect'/de OR 'item response model' OR irt OR rasch OR 'differential item functioning'/de OR dif OR 'computer adaptive testing'/de OR 'item bank' OR 'cross-cultural equivalence')
NOT (('delphi technique'/de OR 'cross-sectional studies'/de OR 'addresses' OR 'biography'/de OR 'case reports' OR 'comment' OR 'directory'/de OR 'editorial'/de OR 'festschrift' OR 'interview'/de OR 'lectures' OR 'legal cases' OR 'legislation'/de OR 'letter'/de OR 'news' OR 'newspaper article' OR 'patient education handout' OR 'popular works' OR 'congresses'/de OR 'consensus development conference'/de OR 'nih consensus development conference' OR 'practice guideline'/de OR NOT ("Prenatal Care" OR "Prenatal Diagnosis" OR "Prenatal Education" OR prenatal OR antenatal OR 'animals'/de))
Filters: English

Total = 10,212
Endnote duplicate and animal removal = 8,585
Rayyan duplicate removal = 8,008

© 2021 Sultan P et al. *JAMA Network Open*.

eAppendix 2. Updated Search July 11, 2020, for Publications of Validated Measures Since Previous Search

PubMed Search –10 results

(("Barkin index of maternal functioning" OR "Inventory of functional status after childbirth" OR "Maternal concerns questionnaire" OR "Postpartum symptom checklist" OR "Rural postpartum QoL" OR "Postpartum QoL" OR "Maternal postpartum QoL tool" OR "Postpartum Quality of life" OR SF36 OR SF12 OR EQ5D OR WHO QoL-BREF OR WHO-DAS II OR Swedish health related QoL OR Nottingham health profile OR "QoL Inventory" OR WHO quality of life-BREF OR Swedish health related quality of life OR "quality of life inventory"))

AND (("Delivery, Obstetric"[Mesh] OR "Labor, Induced"[Mesh] OR "Extraction, Obstetrical"[Mesh] OR "instrumental delivery" OR "vaginal delivery" OR "vaginal birth" OR "vacuum delivery" OR "vacuum assisted delivery" OR "forceps assisted delivery" OR childbirth) AND ("Postpartum Period"[Mesh] OR "Postnatal Care"[Mesh] OR intrapartum OR postpartum OR postnatal OR "Postoperative Complications"[Mesh] OR "Postoperative Care"[Mesh] OR "Postoperative Period"[Mesh] OR "postoperative" OR "post-operative" or "postsurgery" OR "post surgical" OR "postop" OR recovery OR "Recovery Room"[Mesh] OR "recovery room" OR "post-anesthesia" OR "post-anaesthesia" OR "pacu" OR ((post-anesthesia OR post-anaesthesia) AND acute care unit))

NOT ("Prenatal Care"[Mesh] OR "Prenatal Diagnosis"[Mesh] OR "Prenatal Education"[Mesh] OR prenatal OR antenatal))

CINAHL Search – 0 results

(MH "Labor, Induced" OR MH "Vacuum Extraction, Obstetrical" OR MH "Delivery, Obstetric" OR MH "Vaginal Birth" OR "Delivery, Obstetric" OR "Labor, Induced" OR "Extraction, Obstetrical" OR “instrumental delivery” OR “vaginal delivery” OR “vaginal birth” OR “vacuum delivery” OR “vacuum assisted delivery” OR “forceps assisted delivery” OR childbirth) AND (MH "Postnatal Care" OR MH "Postnatal Period" OR MH "Postoperative Period" OR MH "Postoperative Care" OR MH "Post Anesthesia Care" OR "Postpartum Period" OR "Postnatal Care" OR intrapartum OR postpartum OR postnatal OR "Postoperative Complications" OR "Postoperative Care" OR "Postoperative Period" OR “postoperative” OR “post-operative” or “postsurgery” OR “post surgical” OR “postop” OR recovery OR "recovery room" OR “post-anesthesia” OR “post-anaesthesia” OR “pacu” OR ((post-anesthesia OR post-anaesthesia) AND acute care unit)) NOT ("Prenatal Care" OR "Prenatal Diagnosis" OR "Prenatal Education" OR prenatal OR antenatal)

(("Barkin index of maternal functioning" OR "Inventory of functional status after childbirth" OR "Maternal concerns questionnaire" OR "Postpartum symptom checklist" OR "Rural postpartum QoL" OR "Postpartum QoL" OR "Maternal postpartum QoL tool" OR "Postpartum Quality of

© 2021 Sultan P et al. *JAMA Network Open*.

life" OR SF36 OR SF12 OR EQ5D OR WHO QoL-BREF OR WHO-DAS II OR Swedish health related QoL OR Nottingham health profile OR "QoL Inventory" OR WHO quality of life-BREF OR Swedish health related quality of life OR "quality of life inventory"))

Web Of Science Search – 4 results

("Obstetric Delivery" OR "Induced Labor" OR "Obstetrical Extraction " OR “instrumental delivery” OR “vaginal delivery” OR “vaginal birth” OR “vacuum delivery” OR “vacuum assisted delivery” OR “forceps assisted delivery” OR childbirth) AND ("Postpartum Period" OR "Postnatal Care" OR intrapartum OR postpartum OR postnatal OR "Postoperative Complications" OR "Postoperative Care" OR "Postoperative Period" OR “postoperative” OR “post-operative” or “postsurgery” OR “post surgical” OR “postop” OR recovery OR "recovery room" OR “post-anesthesia” OR “post-anaesthesia” OR “pacu” OR ((post-anesthesia OR post-anaesthesia) AND acute care unit)) NOT ("Prenatal Care" OR "Prenatal Diagnosis" OR "Prenatal Education" OR prenatal OR antenatal)

(("Barkin index of maternal functioning" OR "Inventory of functional status after childbirth" OR "Maternal concerns questionnaire" OR "Postpartum symptom checklist" OR "Rural postpartum QoL" OR "Postpartum QoL" OR "Maternal postpartum QoL tool" OR "Postpartum Quality of life" OR SF36 OR SF12 OR EQ5D OR WHO QoL-BREF OR WHO-DAS II OR Swedish health related QoL OR Nottingham health profile OR "QoL Inventory" OR WHO quality of life-BREF OR Swedish health related quality of life OR "quality of life inventory"))

EMBASE Search – 3 results

('delivery, obstetric'/de OR 'labor, induced'/de OR 'extraction, obstetrical'/de OR 'instrumental delivery'/de OR 'vaginal delivery'/de OR 'vaginal birth'/de OR 'vacuum delivery'/de OR 'vacuum assisted delivery'/de OR 'forceps assisted delivery' OR 'childbirth'/de) AND ('postpartum period'/de OR 'postnatal care'/de OR intrapartum OR 'postpartum'/de OR postnatal OR 'postoperative complications'/de OR 'postoperative care'/de OR 'postoperative period'/de OR 'postoperative' OR 'post-operative' OR 'postsurgery' OR 'post surgical' OR 'postop' OR 'recovery'/de OR 'recovery room'/de OR 'post-anesthesia' OR 'post-anaesthesia' OR 'pacu' OR (('post anesthesia' OR 'post anaesthesia') AND acute AND 'care'/de AND 'unit'/de))

(Barkin index of maternal functioning OR "Inventory of functional status after childbirth" OR "Maternal concerns questionnaire" OR "Postpartum symptom checklist" OR "Rural postpartum QoL" OR "rural postpartum quality of life" OR "Postpartum QoL" OR "Maternal postpartum QoL tool" OR "Postpartum Quality of life" OR "Maternal postpartum Quality of Life tool" OR "rural postpartum" OR maternal postpartum tool OR SF36 OR SF12 OR EQ5D OR WHO QoL-BREF OR WHO-DAS II OR Swedish health related QoL OR Nottingham health profile OR QoL Inventory OR who quality of life-BREF OR swedish health related quality of life OR quality of life inventory)

17 total citations

© 2021 Sultan P et al. *JAMA Network Open*.

14 citations after duplicate removal

© 2021 Sultan P et al. *JAMA Network Open*.

Recovery domain (number of individual items within each domain)	Symptoms / items contributing to recovery domain
Physical function (12)	Limited function due to musculoskeletal pathology, cardiovascular deconditioning, ability to perform ADLs, physical independence ability to hold baby, ability to drive, ability to perform heavy lifting, ability to mobilize, ability to sit, abdominal core strength intake of adequate nutrition, ability to exercise
Surgical and medical factors (urology/obstetrical /gynecology/colorectal/ anesthesia/medical) (40)	Urology (urinary symptoms, incontinence, micturition); gynecology (dysmenorrhea, uterine prolapse); colorectal (fecal symptoms, incontinence, hemorrhoids, anal fissure, nausea and vomiting), obstetrical / gynecology (multiple gestation, PV bleeding, lochia, vaginal laceration, incision, cutaneous paresthesia / pruritus/edema, SSI, reduced perineal sensation, retained products of conception, uterine subinvolution, hypertension and pre-eclampsia management, amenorrhea, parity, unplanned delivery mode); colorectal (constipation); medical (infection, fever); medical (carpal tunnel syndrome, thromboembolism, anemia), urology (cystocele); colorectal (rectocele), medical (night sweats, edema, cardiomyopathy), anesthesia (post dural puncture headache, paresthesia)
Pain (18)	Incision / wound; body; muscle, back pain / backache, headache, sensory / affective, pelvic, vaginal, breast, hip, abdominal, requirement for opioids, neck pain / tightness, neuropathic pain
Psychological distress:(50)	
a) Depression	Depression
b) Anxiety	Anxiety
c) Psychological other	Baby blues, general health perception, emotional role, emotional adaption, negative emotional experience, mood, enjoyment of life, fulfillment, mental health disorder, motivation, guilt/worthlessness, helpless/hopelessness, wellbeing, psychomotor retardation, in control, angry, confused, appetite, reduced activity (psychological), stress, life satisfaction, coping behavior, embarrassment, feeling alone, motivation, mourning for delivery mode not achieved, overwhelmed, detached, grief, frustration, fear of becoming pregnant again, tearful, disappointment, PTSD (repeated), loss of self-identity, patience, meeting expectations, feeling of inadequacy, anhedonia, tearful, mental change, impact of coronavirus on mood, suicidal, thoughts about feticide, pain catastrophizing, feeling judged
Psychosocial support (43)	Lifestyle, participation in community activities, social role, interaction with others, social relationships, social function, support from family/staff/friends, psychological support, home / environment adaptation/ maintenance of personal dignity, social isolation, no fixed abode, single mother / donor egg (no spouse), advanced maternal age, access to obstetrician, distance to hospital, transition to returning to work, childcare / nanny issues, change in relationship dynamic with spouse / partner, level of community support, duration of paternity leave, temperament of partner, division of chores, help with overnight childcare chores, change in family dynamic, less personal space, hosting family, connection with partner, older sibling response to new baby, impact of shelter in place due to coronavirus pandemic, cultural variability, language spoken, spirituality e.g. praying, low financial resources, loss of privacy, competition among mothers, domestic violence
Sleep (18)	Sleep quality, latency, sleep quantity, insomnia (middle, terminal), medication requirement, daytime somnolence, sleep disturbance, reduced energy due to lack of sleep, nightmares; number of wake ups per night, influence of sleep on mood, difficulty getting back to sleep after being awoken; sleep quality prior to pregnancy, vivid dreams about baby, sleep deprivation, unpredictable sleep pattern
Fatigue (6)	Mental fatigue, physical fatigue, exhaustion, feeling rested, tiredness, energy levels
Motherhood experience:	
(a) Adapting to maternal role (6)	Caring for baby, parenting, adapting to new role, looking after and interacting with other siblings, caregiving responsibilities, time management
(b) Maternal-neonatal bonding (2)	Bonding, attachment
Infant health (21)	Fetal scalp lacerations, unexpected morbidity, cardiac morbidity, neonatal demise, concern about infant health, concern about infant weight, assistance with nutrition, need for light therapy/antibiotics/CPAP/ECMO, requirement for NICU, prolonged NICU, requirement for repeat

© 2021 Sultan P et al. *JAMA Network Open*.

	hospitalization, requirement for investigations, prematurity, checking repeatedly for breathing, tongue tie, reflux, wind, type of baby (e.g. contented, cries excessively)
Feeding / Breastfeeding Breast health (14)	Breastfeeding, lactation, breast health (mastitis), breastfeeding (latch, milk supply, engorgement, comfort with pumping), confidence in feeding (breast / bottle) breast health (cracked nipples, bleeding nipples), supplementation of breast milk with bottle, perceived success of feeding, time till expression of milk
Sexual function (12)	Behavioral emotive, arousal, physical, partner related, desire, lubrication, orgasm, dyspareunia, sex satisfaction, contraception, sexual health return of sexuality
Appearance / cosmetic (16)	Physical appearance, wound (scar appearance, healing, dehiscence), desire to return to pre-pregnancy weight, wound (stitches, granulation tissue, discharge, inflammation), wound healing, body dysmorphia, stretch marks, dissatisfaction with appearance, hair loss, grooming
Cognition (6)	Memory, critical thinking, problem solving, forgetfulness, decision-making capacity, executive function

eTable 1. Factors Associated With Postpartum Recovery Grouped Into Domains

SSI=surgical site infection, PV=per vagina, ADLs=activities of daily living, PTSD=post-traumatic stress disorder, NICU=neonatal intensive care unit; CPAP=continuous positive airway pressure; ECMO=extracorporeal membrane oxygenation

© 2021 Sultan P et al. *JAMA Network Open*.

eTable 2. Summary of Numbers of Included and Excluded Patient-Reported Outcome Measures in This Review

Validated measures identified in previous review	New measures identified (Source from where identified)	Number of validation studies (Reason for exclusion)
**Obstetric-specific measures**		
Inventory of functional status after childbirth (14)		7 included studies (Exclusions: 3 not validation studies; 2 theses; 2 duplicate data)
Barkin index of maternal functioning (4)	2 (secondary search)	6
Maternal postpartum QoL tool (2)		2
Postpartum symptom checklist (1)		1
Maternal concerns questionnaire (2)		2
Rural postpartum QoL (RPQoL; 1)		1
	Postpartum QoL (found in reference list of RPQoL)	1
Mother generated index		Inconsistent among patients due to free text answering of symptoms (responses not comparable between patients or instruments)
Female sexual distress scale		Measure of sexual function – not a global recovery measure
Sexual function questionnaire medical impact scale		Measure of sexual function – not a global recovery measure
**Non-obstetric specific measures**		
SF-36 (27)		9 included studies (Exclusions: 15 not validation studies; 2 evaluation of single domain; 1 thesis)
SF-12 (8)	1 (secondary search)	7 included studies (Exclusions: 1 evaluation of single domain; 1 not validation study)
EQ5D (9)		8 included studies (Exclusion: 1 not validation study)
WHOQoL-BREF (1)		1
Nottingham health profile (1)		1
WHO-DAS II (1)		1
QoL inventory (1)		1
Swedish HRQoL (1)		1

2 studies evaluated SF-36 and EQ-5D and 1 study evaluated EQ5D and QoL, therefore total number of non-obstetric studies are 26 (not 29).

© 2021 Sultan P et al. *JAMA Network Open*.

eTable 3. Summary of Included Studies

First author, year	Country of study	Language of instrument	Patient number	Response rate	Methodology
**Inventory of Functional Status After Childbirth**					
Aktan, 2010	USA	English	177	73%[d]	Very good
Fawcett, 1988	USA	English	76	Not stated	Very good
McVeigh, 2000[a]	Australia	English	173	71-86%[d]	Very good
McVeigh, 2002[a]	Australia	English	173	Not stated	Very good
Mirghafourvand, 2016	Iran	Persian	200	69%[e]	Very good
Noor, 2015	Malaysia	Malay	108	Not stated	Very good
Norhayati, 2016	Malaysia	Malay	332	42-54%[e]	Very good
**Barkin Index of Maternal Functioning**					
Barkin, 2010	USA	English	31	Not stated	Very good
Barkin, 2014	USA	English	346	Not stated	Adequate
Barkin 2017	USA	English	128	Not stated	Very good
Barkin, 2017	USA	English	24	Not stated	Inadequate
Chamgurdani, 2020	Iran	Not stated	80	100%[e]	Very good
Shamsabi, 2020	Iran	Not stated	530	100%[d]	Very good
**Maternal Postpartum Quality of Life tool**					
Hill, 2006[a]	USA	English	184	78%[d]	Adequate
Hill, 2007[a]	USA	English	184	78%[d]	Inadequate
**Postpartum Symptoms Checklist**					
Maloni, 2005	USA		106	Not stated	Adequate
**Maternal Concerns Questionnaire**					
Sheil, 1995	USA	English	187 (5 theses)	Not stated	Inadequate
Fishbein, 1998	USA	English	100	Not stated	Very good
**Rural Postpartum Quality of Life**					
Huang, 2012	China	Not stated	1,375	Not stated	Inadequate
**Postpartum Quality of Life**					
Nikan, 2016	Iran	Persian	500	100%[f]	Very good
**36-item Short Form Health Survey**					
Bahrami, 2014	Iran	Persian	150	Not stated	Very good
Hoedjes, 2011	Netherlands	Dutch	174	79-82%[e]	Very good
Jansen,[a,b] 2007	Netherlands	Dutch	141	55%[f]	Doubtful
Jansen,[a,b] 2007	Netherlands	Dutch	141	53-65%[e]	Very good
Lagaert, 2017	Belgium	English	109	59-65%[e]	Very good
Lydon-Rochelle, 2001	USA	English	971	50-76%[e]	Very good
Sadat, 2014	Iran	Persian	321	88%[e]	Very good
Torkan, 2009	Iran	Persian	100	77%[e]	Very good
Trivino-Juarez, 2017	Spain	Spanish	546	89%[e]	Very good
**12-item Short Form Health Survey**					
Handa, 2007	USA	English	759	82%[e]	Very good
Hiscock, 2007	Australia	English	328	68-72%[e]	Inadequate
Lin, 2018	Taiwan	Not stated	560	Not stated	Very good
Macarthur, 2016	UK	English	3763	48%[e]	Very good
Nolens, 2018	Uganda	English	646	34-39-%[e]	Inadequate
Norhayati, 2016	Malaysia	Malay	332	47%[f]	Inadequate
Bechard, 2019	France	French	1108	27-40%[e]	Very good
**European Quality of Life-5 Dimensions questionnaire**					
Jansen[a,b], 2007	Netherlands	Dutch	141	55%[f]	Doubtful
Jansen[a,b], 2007	Netherlands	Dutch	141	53-65%[e]	Very good
Kohler, 2018	India	Hindi	224	Not stated	Very good
Nieminen[c], 2016	Sweden	English	56	91%[e]	Very good
Petrou, 2017	UK	English	2161	64%[e]	Very good
Seppanen, 2019	Finland	English	99	35%[e]	Very good
Turkstra, 2013	Australia	English	262	65-97%[d]	Inadequate
Turkstra, 2017	Australia	English	184	54%[e]	Very good
**World Health Organization Quality of Life-BREF score**					
Webster, 2010	Australia	English	221	69%[e]	Very good
**Nottingham health profile**					
Baghirzada, 2013	Canada	English	133	73%[e]	Inadequate
**World Health Organization Disability Assessment Scale-II**					
Senturk, 2012	Ethiopia	English	954	90%[e]	Adequate
**Quality of Life Inventory**					
Nieminen[c], 2016	Sweden	English	56	91%[e]	Very good
**Swedish Health Related Quality of Life**					
Carlander, 2015	Sweden	Swedish	372	68%[e]	Very good

Response rate presented as a range where different groups or time points within the study were reported; methodology reported according to COSMIN risk of bias checklist using a "worst score

© 2021 Sultan P et al. *JAMA Network Open*.

counts" principle and evaluated related to measurement property assessed; a=separate published studies, which likely utilized the same data set; b=studies used to evaluate EQ5D and SF36; c=studies used to evaluate EQ5D and QoL Inventory; d=studies reported response rate but no data of numbers approached / withdrawals and missingness data from patients that completed the survey not presented; e=studies presented response rate and numbers approached and excluded, but not missingness within completed surveys; f=studies reports response rate, patient numbers approached and missing data from completed surveys.

© 2021 Sultan P et al. *JAMA Network Open*.

eTable 4. Risk of Bias Assessment of Methods, Reported Results and Overall Rating of Results From All Included Validation Studies

Instrument	Study author, Year	Methods (risk of bias)	Individual results	Overall rating
**Structural validity**				
IFSAC	Noor, 2015	Very good	RMSEA 0.08 (Malay version)[1]	-
Barkin	Barkin, 2014	Adequate	CFA not reported. Eigen values 5.83 and 1.55 for 2 factors[2]	?
Maternal postpartum QoL tool (MAPPQoL)	Hill, 2006	Adequate	CFA not reported. Eigen values ≥1 for 8 components[3]	?
Postpartum symptom checklist		Inadequate	Not assessed	?
Maternal concerns questionnaire	Sheil, 1995	Inadequate	46 item questionnaire became 50 items without analysis of structural validity[4]	?
RPQoL	Huang, 2012	Very good	CFA performed RMSEA=0.05[5]	+
PQoL	Nikan, 2016	Very good	CFA results from the theoretical model showed an acceptable fit for each factor and the scale RMSEA 0.04[6]	+
SF-36		Not assessed	Not assessed	?
SF-12		Not assessed	Not assessed	?
EQ5D		Not assessed	Not assessed	?
WHOQoL-BREF		Not assessed	Not assessed	?
Nottingham Health Profile		Not assessed	Not assessed	?
WHO disability assessment scale II		Not assessed	Not assessed	?
QoL inventory		Not assessed	Not assessed	?
Swedish HRQoL		Not assessed	Not assessed	?
**Internal consistency**				
IFSAC	Noor, 2015<br>McVeigh, 2000<br>Aktan, 2010<br>Mirghafourvand, 2016<br>Mcveigh, 2002<br>Fawcett, 1988	Very good<br>Very good<br>Very good<br>Very good<br>Very good<br>Very good	Cronbach alpha <0.7 in ¼ dimensions[1],<br>0.9 in Australian cohort[7]<br>0.8 in USA cohort[8]<br>0.73 in Iranian cohort[9]<br>Cronbach alpha <0.7 in 3/5 dimensions at weeks 6, 12 and 2/5 at 24 weeks[10]<br>Cronbach alpha 0.76[11]	+<br>+<br>+<br>+<br>-<br>+
Barkin	Barkin, 2010<br>Barkin, 2014<br>Barkin, 2017<br>Chamgurdani, 2020<br>Shamasbi, 2020	Very good<br>Very good<br>Very good<br>Very good<br>Very good	Cronbach alpha 0.87 at 4-6 weeks[12]<br>Cronbach alpha 0.87 at 4-6 weeks[2]<br>Cronbach alpha 0.83 at 6 weeks[13]<br>Cronbach alpha 0.88[14]<br>Cronbach alpha 0.88[15]	+<br>+<br>+<br>+<br>+
Maternal postpartum QoL tool (MAPPQoL)	Hill, 2006	Very good	Cronbach alpha 0.96 (>0.7 for all 5 domains)[3]	+
Postpartum symptom checklist	Maloni, 2005	Very good	Cronbach alpha 0.79-0.84[16]	+
Maternal concerns questionnaire	Sheil, 1995<br>Fishbein, 1998	Very good<br>Very good	Cronbach alpha 0.9[4]<br>Cronbach Alpha 0.95[17]	+<br>+
RPQoL	Huang, 2012	Very good	Cronbach alpha 0.87[5]	+
PQoL	Nilan, 2016	Very good	Cronbach alpha 0.88[6]	+
SF-36	Jansen, 2007<br>Jansen, 2007	Very good<br>Very good	All Cronbach alpha >0.7 (except 2 subset values [18])<br>All Cronbach alpha >0.7 (except 1 subset value[19])	+<br>+
SF-12		Not assessed	Not assessed	?
EQ5D		Not assessed	Not assessed	?
WHOQoL-BREF	Webster, 2010	Very good	Cronbach alpha ≥0.7 for all domains[20]	+
Nottingham Health Profile	Baghirzada, 2013	Very good	Cronbach's alpha 0.6-0.8 for each dimension 1-2 weeks (>0.7 in 5/6 dimensions)[21]	+

© 2021 Sultan P et al. *JAMA Network Open*.

WHO disability assessment scale-II		Not assessed	Not assessed	?
QoL inventory		Not assessed	Not assessed	?
Swedish HRQoL		Not assessed	Not assessed	?
**Cross cultural validity / Measurement invariance**				
IFSAC		Not assessed	Not assessed	?
Barkin	Barkin, 2017	Inadequate	Cognitive interviews with underserved obstetric population- qualitative reporting (no multiple group factor analysis or DIF performed)[22]	?
Maternal postpartum QoL tool (MAPPQoL)		Not assessed	Not assessed	?
Postpartum symptom checklist		Not assessed	Not assessed	?
Maternal concerns questionnaire		Not assessed	Not assessed	?
RPQoL	Huang, 2012	Not assessed	Rural China scores not compared to other population[5]. English translation not available	?
PQoL	Nikan, 2016	Not assessed	Not assessed (some questions seem not applicable to the western population e.g concern regarding pollution, worry about child having an accident. Iranian version validated)[6]	?
SF-36		Not assessed	Not assessed	?
SF-12		Not assessed	Not assessed	?
EQ5D		Not assessed	Not assessed	?
WHOQoL-BREF		Not assessed	Not assessed	?
Nottingham Health Profile		Not assessed	Not assessed	?
WHO disability assessment scale-II	Senturk, 2012	Not assessed	Not assessed. Questionable relevance to western population as Ethiopian study[23]	?
QoL inventory		Not assessed	Not assessed	?
Swedish HRQoL		Not assessed	Not assessed	?
**Reliability**				
IFSAC	Noor, 2015<br>McVeigh, 2002<br>Mirghafourvand, 2016<br>Fawcett, 1988	Very good<br>Very good<br>Very good<br>Very good	ICC <0.7 in 1 dimension [1]<br>ICC <0.7[10]<br>ICC 0.96[9]<br>Test-retest ICC 0.86[11] (<0.7 in 1 dimension)<br>Subscale score to total score<0.7[11]	+<br>-<br>+<br>+<br>-
Barkin	Barkin, 2014<br>Chamgurdani, 2020<br>Shamasbi, 2020	Very good<br>Very good<br>Very good	ICC ranged from 0.5-0.7 at 4-6 weeks[2]<br>ICC 0.8[14]<br>ICC 0.85[15]	-<br>+<br>+
Maternal postpartum QoL tool (MAPPQoL)	Hill, 2007<br>Hill, 2006	Very good<br>Very good	ICC ranged from 0.47-0.9[24]<br>Test-retest reliability 0.74 within 2 week interval[3]	-<br>+
Postpartum symptom checklist		Not assessed	Not assessed	?
Maternal concerns questionnaire		Not assessed	Not assessed	?
RPQoL		Not assessed	Not assessed	?
PQoL	Nikan, 2016	Very good	ICC 0.87[6]	+
SF-36	Jansen, 2007	Doubtful	Not assessed (ICC or weighted kappa not reported)[18]	?
SF-12		Not assessed	Not assessed	?
EQ5D		Not assessed	Not assessed	?
WHOQoL-BREF	Webster, 2010	Very good	ICC 0.2-0.8 at 6 weeks[20]	-
Nottingham Health Profile		Not assessed	Not assessed	?
WHO disability assessment scale-II	Senturk, 2012	Not assessed	Not assessed (although authors report high number of women scoring 0 suggesting high ceiling effects)[23]	?
QoL inventory		Not assessed	Not assessed	?
Swedish HRQoL		Not assessed	Not assessed	?
**Measurement error**				
IFSAC		Not assessed	Not assessed	?

© 2021 Sultan P et al. *JAMA Network Open*.

Barkin		Not assessed	Not assessed	?
Maternal postpartum QoL tool (MAPPQoL)		Not assessed	Not assessed	?
Postpartum symptom checklist		Not assessed	Not assessed	?
Maternal concerns questionnaire		Not assessed	Not assessed	?
RPQoL		Not assessed	Not assessed	?
PQoL		Not assessed	Not assessed	?
SF-36		Not assessed	Not assessed	?
SF-12		Not assessed	Not assessed	?
EQ5D		Not assessed	Not assessed	?
WHOQoL-BREF		Not assessed	Not assessed	?
Nottingham Health Profile		Not assessed	Not assessed	?
WHO disability assessment scale-II		Not assessed	Not assessed	?
QoL inventory		Not assessed	Not assessed	?
Swedish HRQoL		Not assessed	Not assessed	?
**Hypothesis testing (comparison of scores between measures / plausible group comparisons)**				
IFSAC		Very good	Higher scores in women at 1 and 6 months in the absence of severe maternal morbidity (2.2 vs 2.3)[25]	+
Barkin	Barkin, 2014	Very good	Correlation with Gratification Checklist (r= 0.53) and mental functioning component of SF-12 (r=0.32) and with Hamilton rating scale for depression (r=-0.26)[2]<br>Correlated with SF-12 mental dimension (r=0.32)<br>but not SF-12 physical (r=0.06)[2]	+<br>+<br>-
	Barkin, 2010	Very good	Correlation with Gratification Checklist (r=0.56) and mental functioning component of SF-12 (r=0.39) and with Hamilton rating scale for depression (r=-0.21)[12]	+
	Barkin, 2017	Very good	Inverse correlation between score and depressive symptoms measured by EPDS (r=-0.45)[13]	+
	Chamgurdani, 2020	Very good	Higher scores without depression (mean difference 30 (95% CI 24-36)[14]<br>Correlation with depression score (r=-0.79)	+<br>+
	Shamasbi, 2020	Very good	Correlation between scores and mental health inventory scores (r=0.2-0.6)[15]	+
Maternal postpartum QoL tool (MAPPQoL)	Hill, 2006	Adequate (unclear if comparator instruments appropriate (e.g. Overall satisfaction with Life)	Correlation r=0.7 with satisfaction score and significant correlation (though r<0.7) with sleep score, and mood subscales of MAACL-R (Multiple affect adjective check list-revised)[3]	+<br>++
	Hill, 2007	Very good	Lower overall scores in pre-term and term groups, but not statistically significant[24]	-
Postpartum symptom checklist	Maloni, 2005	Adequate	Mean number of symptoms with SVD lower than with CD[16] but unclear if Likert applied to presence of symptom severity	?
Maternal concerns questionnaire	Sheil, 1995<br>Fishbein, 1998	Not assessed	Not assessed in 1 study[4] and descriptive data in other study[17]	?
RPQoL	Hunag, 2012	Very good	No differences between SVD and CD at 0-3, 4-6 or 7-12 months[5]	-
PQoL	Nikan, 2016	Very good	Moderate correlation to SF-12 (r=0.5)[6]<br>Total score higher after SVD vs CD and in women with PPD vs no PPD at week 8[6]	+<br>+
SF-36	Jansen, 2007	Very good	Worse after emergency CD vs SVD at week 1 (range in means 8-27 between different dimensions)[18]	+

© 2021 Sultan P et al. *JAMA Network Open*.

	Lydon-Rochelle, 2001	Very good	Worse after CD vs SVD at 5 weeks (range in means 4-12 between different dimensions)[26]	+
	Hoedjes, 2011	Very good	Worse after severe pre-eclampsia in all domains at 6 weeks and<br>60% domains at 12 weeks (range in means 4-15 between different dimensions)[27]	+<br>+
	Sadat, 2014	Very good	90% of dimensions worse in women with PPD vs no PPD at 8 weeks and 16 weeks.[28]	+
	Torkan 2009	Very good	No difference between SVD and CD at 6-8 weeks in 6/8 domains[29] and 12-14 weeks in 7/8 domains	-
SF-12	Handa, 2007	Very good	Urinary or fecal incontinence associated with worse scores at 6 months for ½ domains [30]	-
	Lin, 2018	Very good	At 3 months and 12 months, women with urinary incontinence after CD and SVD had no difference in both domains [31]	-, -
	MacArthur, 2016	Very good	Persistent urinary incontinence associated with worse scores up to 12 years post all delivery modes[32]	+
	Nolens, 2018	Very good	Vacuum associated with worse scores in ½ domains compared with CD at 6 weeks,<br>no difference at 6 months[33]	-<br>-
	Norhayati, 2017	Very good	No difference at 1 and 6 months in women experiencing severe maternal morbidity vs no morbidity[34]	-
	Bechard, 2019	Very good	No difference at 3 months, ½ domains better at 12 months following SVD compared to CD[35]	-
EQ5D	Petrou, 2017	Very good	Higher score 12 months after SVD compared to CD[36]	+
	Seppanen, 2019	Very good	Same score (in 68-96% for each domain) following ITU stay at 6 months (19% worsened score)[37]	-
	Kohler, 2018	Very good	Worse after CD vs SVD and<br>worse after episiotomy up to 30 days[38]	+<br>+
	Jansen, 2007	Very good	Worse after emergency CD up to 6 weeks[18].<br>3 week scores higher after SVD vs elective CD[19]	+<br>+
WHOQoL-BREF	Webster, 2010	Very good	Worse scores in post-natal depression vs no postnatal depression groups at 6 weeks[20]<br>6 weeks correlation between WHOQoL-BREF and personal well-being domains 0.35-0.7, satisfactory (>0.45) in the majority of domains [20]	+<br>+
Nottingham Health Profile	Baghirzada, 2013	Very good	2/6 dimensions score worse with CD vs SVD at 1-2 weeks[21]<br>None of the dimensions correlated strongly (r≥0.7 or moderately >0.45) with WHOQoLBREF dimensions[21]	-<br>-
WHO disability assessment scale-II	Senturk, 2012	Adequate	PHQ-13 (patient health questionnaire) and SRQ-20 (self-reporting questionnaire) associated with WHODAS-II score at 2 months[23] (correlation not reported as r)<br>WHODAS score associated with maternal ill health[23] (correlation not reported as r)	+<br>+
QoL inventory		Not assessed	Not assessed	?

© 2021 Sultan P et al. *JAMA Network Open*.

Swedish HRQoL	Carlander, 2015	Very good (though questionable hypothesis)	3/16 domains were different between delivery modes at 5 yrs postpartum[39]	?
**Responsiveness**				
IFSAC	McVeigh, 2000<br><br>Norhayati, 2016	Very good<br><br>Very good	3/5 dimensions improve from 6, 12 and 24 weeks [7]<br><br>Higher overall scores between 1 and 6 months.<br>Only 1 dimension worsened from 1 to 6 months[25]	+<br><br>+<br>+
Barkin		Not assessed	Not assessed	?
Maternal postpartum QoL tool (MAPPQoL)	Hill, 2007<br><br>Hill, 2006	Inadequate<br><br>Very good	Qualitative increase from week 1 to week 3, statistics not performed to confirm[24]<br><br>Increase from week 1 to week 3,[3] likely to be the same data set as other study	?<br><br>+
Postpartum symptom checklist	Maloni, 2005	Adequate	Number of symptoms decreased between day 2 and weeks 1, 2, 3, 4 , 5, 6 (ANOVA) but unclear if this is how the PSC should be reported (unclear if scored as 1 score per symptom[16] as severity also reported in this study so unclear Likert scoring)	?
Maternal concerns questionnaire		Not assessed	Not assessed	?
RPQoL	Huang, 2012	Inadequate (study did not seek to investigate responsiveness)	Not formally assessed with statistical analysis. Qualitative improvement between 0-3 and 4-6 months but not change between 4-6 and 7-12 months[5]	?
PQoL	Nikan, 2016	Not assessed	Only 8 week assessment performed [6]	?
SF-36	Jansen, 2007<br><br>Lagaert, 2017<br><br>Hoedjes, 2011<br><br>Bahrami, 2014<br>Trivino-Juarez, 2017	Very good<br><br>Very good<br><br>Very good<br><br>Very good<br>Very good	All domains of SF-36 except General Health significantly increased between week 1 and 6)[18]<br><br>No change between week 6 and 6 months[40]<br><br>Improvement between 6-12 weeks in pre-eclamptic women[27]<br><br>Improvement in scores between 6-8 weeks and 12-14 weeks[41]<br>No change between 6 weeks and 6 months in >75% subgroups for all delivery modes except forceps delivery[42]	+<br><br>-<br><br>+<br><br>+<br>-
SF-12	Hiscock, 2007<br><br>Nolens, 2018<br><br>Norhayati, 2016<br><br>Bachard, 2019	Inadequate<br><br>Inadequate<br><br>Inadequate (no studies sought to investigate responsiveness)<br><br>Inadequate	Similar scores at 10 and 12 months but no statistical analysis[43]<br><br>Increased scores between 6 weeks and 6 months but no statistical analysis [33]<br><br>Increased scores between 1 and 6 months but no statistical analysis[34]<br><br>No statistical analysis. Physical qualitatively improves whereas mental domain decreases from month 3 to 12[35]	?<br><br>?<br><br>?<br><br>?
EQ5D	Turkstra, 2017<br><br>Turkstra, 2013<br><br>Nieminen, 2016<br><br>Kohler, 2018<br><br>Jansen, 2007	Very good<br><br>Inadequate<br><br>Very good<br><br>Very good<br><br>Very good	No change in total score between 2nd trimester and 6 weeks[44] (1 dimension improved)<br><br>No statistics performed. Qualitative increase from 6 weeks to 6 months and decrease from 6 months to 1 year[45]<br><br>No change up to 8 weeks [46]<br><br>Improved between 0-3; 3-7 and 21-30 days[38]<br><br>Improvement over weeks 1, 3 and 6 after all delivery modes[18]	-<br><br>?<br><br>-<br><br>+, +<br><br>+
WHOQoL BREF		Not assessed	Not assessed	?

© 2021 Sultan P et al. *JAMA Network Open*.

Nottingham Health Profile	Baghirzada, 2013	Inadequate (studies did not seek to investigate responsiveness)	Improved scores from 1-2 days to 1-2 weeks in 5/6 domains following SVD and 4/6 following CD, but no statistical analysis [21]	?
WHO disability assessment scale-II	Senturk, 2012	Not assessed	Not assessed ((2 Postpartum time points not assessed) but scores improved from pregnancy to 8 weeks postpartum)[23]	?
QoL inventory	Nieminen, 2016	Very good	No change in score from week 1 to week 8[46]	-
Swedish HRQoL		Not assessed	Not assessed	?

IFSAC=Inventory of Functional Status After Childbirth; Barkin=Barkin Index of Maternal Functioning; PSC=Postpartum Symptom Checklist; RPQoL=Rural postpartum Quality of Life; PQoL=Postpartum Quality of Life; SF-36=36-item Short Form Health Survey; SF-12=12-item Short Form Health Survey; EQ5D=European Quality of Life-5 Dimensions questionnaire; WHOQoL-BREF=World Health Organization Quality of Life-BREF score; Nottingham HP=Nottingham Health Profile; WHO DAS-II =World Health Organization disability assessment scale- II; QoL=Quality of Life; HRQoL=Health related quality of life;
CFA= confirmatory factor analysis; RMSEA=root mean square error of approximation; DIF=differential item functioning; ICC=intraclass correlation coefficient; SVD=spontaneous vaginal delivery; CD=cesarean delivery; PPD=postpartum depression; ITU=Intensive Care
Methods rating: Very good, Adequate, Doubtful, Inadequate or Not assessed
Overall rating: Sufficient (+), Insufficient (-), Inconsistent (+/-) or Indeterminate (?)

© 2021 Sultan P et al. *JAMA Network Open*.

eTable 5. Summary of Ratings of Overall Psychometric Measurement Properties From Grouped Studies for Each Measure and Grade Assessment of Level of Evidence

Instrument	Study author, Year	Summary or pooled result	Overall rating	GRADE Quality of evidence
**Structural validity**				
IFSAC	Noor, 2015	RMSEA 0.08 (Malay version)[1]	-	Moderate (downgraded as no response rate or missingness reported)
Barkin	Barkin, 2014	CFA not reported. Eigen values 5.83 and 1.55 for 2 factors[2]	?	Very low (no response rate or missingness data)
Maternal postpartum QoL tool (MAPPQoL)	Hill, 2006	CFA not reported. Eigen values ≥1 for 8 components[3]	?	Very low
Postpartum symptom checklist		Not assessed	?	Very low
Maternal concerns questionnaire	Sheil, 1995	46 item questionnaire became 50 items without analysis of structural validity[4]	?	Very low (no response rates or missingness data, data from theses and convenience samples and following refinement only 8 people reviewed the instrument – 5 mothers and 3 nurses)
RPQoL	Huang, 2012	CFA performed RMSEA=0.05[5]	+	Moderate (likely attrition / selection bias, unknown response rate and generalizability outside rural China)
PQoL	Nikan, 2016	CFA results from the theoretical model showed an acceptable fit for each factor and the scale RMSEA 0.04[6]	+	High
SF-36		Not assessed	?	Very low (not assessed)
SF-12		Not assessed	?	Very low (not assessed)
EQ5D		Not assessed	?	Very low (not assessed)
WHOQoL-BREF		Not assessed	?	Very low (not assessed)
Nottingham Health Profile		Not assessed	?	Very low (not assessed)
WHO disability assessment scale		Not assessed	?	Very low (not assessed)
QoL inventory		Not assessed	?	Very low (not assessed)
Swedish HRQoL		Not assessed	?	Very low (not assessed)
**Internal consistency**				
IFSAC	Noor, 2015<br>McVeigh, 2000<br>Aktan, 2010<br>Mirghafourvand, 2016<br>Mcveigh, 2002<br>Fawcett, 1988	Cronbach alpha <0.7 in ¼ dimensions[1],<br>0.9 in Australian cohort[7]<br>0.8 in USA cohort[8]<br>0.73 in Iranian cohort[9]<br>Cronbach alpha <0.7 in 3/5 dimensions at weeks 6, 12 and 2/5 at 24 weeks[10]<br>Cronbach alpha 0.76[11]	+	Moderate (Malay, Persian and English versions assessed[1][10][9]; response rate not stated in 3 studies; 1 good response rate and 1 borderline response rate)
Barkin	Barkin, 2010<br>Barkin, 2014	Cronbach alpha 0.87 at 4-6 weeks[12]<br>Cronbach alpha 0.87 at 4-6 weeks[2]	+	Low (downgraded due to no reporting of response rates or missing data, attrition bias and convenience sampling)

© 2021 Sultan P et al. *JAMA Network Open*.

	Barkin, 2017<br><br>Chamgurdani, 2020<br><br>Shamasbi, 2020	Cronbach alpha 0.83 at 6 weeks[13]<br><br>Cronbach alpha 0.88[14]<br><br>Cronbach alpha 0.88[15]		
Maternal postpartum QoL tool (MAPPQoL)	Hill, 2006	Cronbach alpha 0.96 (>0.7 for all 5 domains)[3]	+	Low (downgraded due to convenience sampling; no missingness or numbers of eligible women)
Postpartum symptom checklist	Maloni, 2005	Cronbach alpha 0.79-0.84[16]	+	Low (no response rate or missing data reported, convenience sample)
Maternal concerns questionnaire	Sheil, 19, 1995<br><br>Fishbein, 1998	Cronbach alpha 0.9[4]<br><br>Cronbach Alpha 0.95[17]	+	Low (data from theses in 1 study rather than peer reviewed published studies and convenience samples in both studies, response rates and missing data not reported in either study)
RPQoL	Huang, 2012	Cronbach alpha 0.87[5]	+	Moderate (unknown response rate and survey completion in rural China)
PQoL	Nilan, 2016	Cronbach alpha 0.88[6]	+	High
SF-36	Jansen, 2007<br><br>Jansen, 2007	All Cronbach alpha >0.7 (except 2 subset values [18])<br><br>All Cronbach alpha >0.7 (except 1 subset value[19])	+	Moderate (two good quality studies but downgraded due to 1 study not reporting missingness data and low response rates)
SF-12		Not assessed	?	Very low (not assessed)
EQ5D		Not assessed	?	Very low (not assessed)
WHOQoL-BREF	Webster, 2010	Cronbach alpha ≥0.7 for all domains[20]	+	Moderate (no report of missingness data; borderline response rate)
Nottingham Health Profile	Baghirzada, 2013	Cronbach's alpha 0.6-0.8 for each dimension 1-2 weeks (>0.7 in 5/6 dimensions)[21]	+	Moderate (no report of missingness data)
WHO disability assessment scale-II		Not assessed	?	Very low (not assessed)
QoL inventory		Not assessed	?	Very low (not assessed)
Swedish HRQoL		Not assessed	?	Very low (not assessed)
**Cross cultural validity / Measurement invariance**				
IFSAC		Not assessed	?	Very low
Barkin	Barkin, 2017	Cognitive interviews with underserved obstetric population-qualitative reporting (no multiple group factor analysis or DIF performed)[22]	?	Very low (no reporting of response rate or missingness data)
Maternal postpartum QoL tool (MAPPQoL)		Not assessed	?	Very low (not assessed)
Postpartum symptom checklist		Not assessed	?	Very low (not assessed)
Maternal concerns questionnaire		Not assessed	?	Very low (not assessed)
RPQoL	Huang, 2012	Rural China scores not compared to other population[5]. English translation not available	?	Very low (not assessed)
PQoL	Nikan, 2016	Not assessed (some questions seem not applicable to the western population e.g concern regarding pollution, worry about child having an accident. Iranian version validated)[6]	?	Very low (not assessed)
SF-36		Not assessed	?	Very low (studies did not assess)
SF-12		Not assessed	?	Very low (not assessed)
EQ5D		Not assessed	?	Very low (not assessed)
WHOQoL-BREF		Not assessed	?	Very low (not assessed)

© 2021 Sultan P et al. *JAMA Network Open*.

Nottingham Health Profile		Not assessed	?	Very low (not assessed)
WHO disability assessment scale-II	Senturk, 2012	Not assessed. Questionable relevance to western population as Ethiopian study[23]	?	Very low (not assessed)
QoL inventory		Not assessed	?	Very low (not assessed)
Swedish HRQoL		Not assessed	?	Very low (not assessed)
**Reliability**				
IFSAC	Noor, 2015<br><br>McVeigh, 2002<br><br>Mirghafourvand, 2016<br><br>Fawcett, 1988	ICC <0.7 in 1 dimension [1]<br><br>ICC <0.7[10]<br><br>ICC 0.96[9]<br><br>Test-retest ICC 0.86[11] (<0.7 in 1 dimension)<br>Subscale score to total score<0.7[11]	+	Low (downgraded due unclear response rates and no missingness data in 3 studies; inadequate response rate in 1 study; Malay and Persian versions included)
Barkin	Barkin, 2014	Inter-item correlations ranged from 0.5-0.7 4-6 weeks[2]<br><br>ICC 0.8[14]<br><br>ICC 0.85[15]	+	Low (no response rate in 1 study, unclear eligible and withdrawals and no missingness data in studies)
Maternal postpartum QoL tool (MAPPQoL)	Hill, 2007<br><br>Hill, 2006	Inter-item correlations ranged from 0.47-0.9[24]<br><br>Test-retest reliability 0.74 within 2 week interval[3]	+/-	Moderate (no missingness data or numbers of women approached)
Postpartum symptom checklist		Not assessed	?	Very low (not assessed)
Maternal concerns questionnaire		Not assessed	?	Very low (not assessed)
RPQoL		Not assessed	?	Very low (not assessed)
PQoL	Nikan, 2016	ICC 0.87[6]	+	High
SF-36	Jansen, 2007	Not assessed (ICC or weighted kappa not reported)[18]	?	Very low (not assessed)
SF-12		Not assessed	?	Very low (not assessed)
EQ5D		Not assessed	?	Very low (not assessed)
WHOQoL-BREF	Webster, 2010	ICC 0.2-0.8 at 6 weeks[20]	-	Moderate (missingness data not presented)
Nottingham Health Profile		Not assessed	?	Very low (not assessed)
WHO disability assessment scale-II	Senturk, 2012	Not assessed (although authors report high number of women scoring 0 suggesting high ceiling effects)[23]	?	Very low
QoL inventory		Not assessed	?	Very low (not assessed)
Swedish HRQoL		Not assessed	?	Very low (not assessed)
**Measurement error**				
IFSAC		Not assessed	?	Very Low (no studies which assessed this)
Barkin		Not assessed	?	Very low (not assessed)
Maternal postpartum QoL tool (MAPPQoL)		Not assessed	?	Very low (not assessed)
Postpartum symptom checklist		Not assessed	?	Very low (not assessed)
Maternal concerns questionnaire		Not assessed	?	Very low (not assessed)

© 2021 Sultan P et al. *JAMA Network Open*.

RPQoL		Not assessed	?	Very low (not assessed)
(PQoL		Not assessed	?	Very low (not assessed)
SF-36		Not assessed	?	Very Low (no studies which assessed this)
SF-12		Not assessed	?	Very low (not assessed)
EQ5D		Not assessed	?	Very low (not assessed)
WHOQoL-BREF		Not assessed	?	Very low (not assessed)
Nottingham Health Profile		Not assessed	?	Very low (not assessed)
WHO disability assessment scale-II		Not assessed	?	Very low (not assessed)
QoL inventory		Not assessed	?	Very low (not assessed)
Swedish HRQoL		Not assessed	?	Very low (not assessed)
**Hypothesis testing (comparison of scores to other measures / plausible group comparisons)**				
IFSAC		Higher scores in women at 1 and 6 months in the absence of severe maternal morbidity (2.2 vs 2.3)[25]	+	Low (high attrition rate)
Barkin	Barkin, 2014<br><br><br><br><br><br>Barkin, 2010<br><br><br><br>Barkin, 2017<br><br><br>Chamgurdani, 2020<br><br><br>Shamasbi, 2020	Correlation with Gratification Checklist (r= 0.53) and mental functioning component of SF-12 (r=0.32) and with Hamilton rating scale for depression (r=-0.26)[2]<br><br>Correlated with SF-12 mental dimension (r=0.32) but not SF12 physical (r=0.06)[2]<br><br>Correlation with Gratification Checklist (r=0.56) and mental functioning component of SF-12 (r=0.39) and with Hamilton rating scale for depression (r=-0.21)[12]<br><br>Inverse correlation between score and depressive symptoms measured by EPDS (r=-0.45)[13]<br><br>Higher scores without depression (mean difference 30 (95% CI 24-36)[14]<br>Correlation with depression score (r=-0.79)<br><br>Correlation between scores and mental health inventory scores (r=0.2-0.6)[15]	+	Low (downgraded due to unclear methodology of when survey completed, unclear withdrawals / response rates/ missingness among completed surveys and convenience sampling)
Maternal postpartum QoL tool (MAPPQoL)	Hill, 2006<br><br><br><br>Hill, 2007	Correlation r=0.7 with satisfaction score and significant correlation (though r<0.7) with sleep score, and mood subscales of MAACL-R (Multiple affect adjective check list-revised)[3]<br><br>Lower overall scores in pre-term and term groups, but not statistically significant[24]	+	Very Low (same data set used in both studies, analyzed in different ways; numbers eligible and missingness data not presented)
Postpartum symptom checklist	Maloni, 2005	Mean number of symptoms with SVD lower than with CD[16] but unclear if Likert applied to presence of symptom severity	?	Low (attrition bias response rate not presented, and convenience sample)
Maternal concerns questionnaire	Sheil, 1995<br>Fishbein, 1998	Not assessed-no plausible hypothesis[4] in 1 study and descriptive data in other study[17]	?	Very low (not assessed)

© 2021 Sultan P et al. *JAMA Network Open*.

RPQoL	Hunag, 2012	No differences between SVD and CD at 0-3, 4-6 or 7-12 months[5]	-	Moderate (response rate not stated, unclear response rate)
PQoL	Nikan, 2016	Moderate correlation to SF-12 (r=0.5)[6]<br>Total score higher after SVD vs CD and in women with PPD vs no PPD at week 8[6]	+	Moderate (downgraded due to inconsistency)
SF-36	Jansen, 2007<br><br>Lydon-Rochelle, 2001<br><br>Hoedjes, 2011<br><br>Sadat, 2014<br><br>Torkan 2009	Worse after emergency CD vs SVD at week 1 (range in means 8-27 between different dimensions)[18]<br><br>Worse after CD vs SVD at 5 weeks (range in means 4-12 between different dimensions)[26]<br><br>Worse after severe pre-eclampsia in all domains at 6 weeks and 60% domains at 12 weeks (range in means 4-15 between different dimensions)[27]<br><br>90% of dimensions worse in women with PPD vs no PPD at 8 weeks and 16 weeks.[28]<br><br>No difference between SVD and CD at 6-8 weeks in 6/8 domains[29] and 12-14 weeks in 7/8 domains	+	Low (downgraded due to missingness data lacking in 4/5 studies; low response rates, lack of hypotheses stated by authors of included studies and due to inconsistency in differences demonstrated beyond 6 weeks)
SF-12	Handa, 2007<br><br>Lin, 2018<br><br>MacArthur, 2016<br><br>Nolens, 2018<br><br>Norhayati, 2017<br><br>Bechard, 2019	Urinary or fecal incontinence associated with worse scores at 6 months for ½ domains [30]<br><br>At 3 months and 12 months, women with urinary incontinence after CD and SVD had no difference in both domains [31]<br><br>Persistent urinary incontinence associated with worse scores up to 12 years post all delivery modes[32]<br><br>Vacuum associated with worse scores in ½ domains compared with CD at 6 weeks, no difference at 6 months[33]<br><br>No difference at 1 and 6 months in women experiencing severe maternal morbidity vs no morbidity[34]<br><br>No difference at 3 months, ½ domains better at 12 months following SVD compared to CD[35]	-	Low (low response rate and missingness data not reported in in most studies)
EQ5D	Petrou, 2017<br><br>Seppanen, 2019<br><br>Kohler, 2018<br><br>Jansen, 2007	Higher score 12 months after SVD compared to CD[36]<br><br>Same score (in 68-96% for each domain) following ITU stay at 6 months (19% worsened score)[37]<br><br>Worse after CD vs SVD and worse after episiotomy up to 30 days[38]<br><br>Worse after emergency CD up to 6 weeks[18]. 3 week scores higher after SVD vs elective CD[19]	+	Low (low response rate in 3 studies, no response rate reported in 1 study, missing data not reported in any studies)
WHOQoL-BREF	Webster, 2010	Worse scores in post-natal depression vs no postnatal depression groups at 6 weeks[20]	+	Moderate (downgraded due to no missingness data, selection bias / attrition bias)

© 2021 Sultan P et al. *JAMA Network Open*.

		6 weeks correlation between WHOQoL-BREF and personal well-being domains 0.35-0.7[20]		
Nottingham Health Profile	Baghirzada, 2013	2/6 dimensions score worse with CD vs SVD at 1-2 weeks[21]<br>None of the dimensions correlated strongly (r≥0.7) with WHOQoLBREF dimensions[21]	-	Moderate (downgraded due to no missingness data presented)
WHO disability assessment scale-II	Senturk, 2012	PHQ-13 (patient health questionnaire) and SRQ-20 (self-reporting questionnaire) associated with WHODAS score at 2 months[23] (correlation not reported as r)<br>WHODAS score associated with maternal ill health[23] (correlation not reported as r)	+	Low (down-graded due to statistical methods-use of Multiplier values to report effect, no missingness data)
QoL inventory		Not assessed	?	Very low (not assessed)
Swedish HRQoL	Carlander, 2015	3/16 domains were different between delivery modes at 5 yrs postpartum[39]	?	Low
**Responsiveness**				
IFSAC	McVeigh, 2000<br><br>Norhayati, 2016	3/5 dimensions improve from 6, 12 and 24 weeks [7]<br><br>Higher overall scores between 1 and 6 months. 1 dimension worsens from 1 to 6 months[25]	+	Low (downgraded due to response rate and no missingness data presented)
Barkin		Not assessed	?	Very low (not assessed)
Maternal postpartum QoL tool (MAPPQoL)	Hill, 2007<br><br>Hill, 2006	Qualitative increase from week 1 to week 3, statistics not performed to confirm[24]<br><br>Increase from week 1 to week 3,[3] likely to be the same data set as other study	?	Low (no missingness data or numbers of eligible women, and analyses from 2 studies appear to be from same data set)
Postpartum symptom checklist	Maloni, 2005	Number of symptoms decreased between day 2 and weeks 1, 2, 3, 4 , 5, 6 (ANOVA) but unclear if this is how the PSC should be reported (unclear if scored as 1 score per symptom[16] as severity also reported in this study so unclear Likert scoring)	?	Low (response rate, missingness, numbers eligible not stated)
Maternal concerns questionnaire		Not assessed	?	Very low (not assessed)
RPQoL	Huang, 2012	Not formally assessed with statistical analysis. Qualitative improvement between 0-3 and 4-6 months but not change between 4-6 and 7-12 months[5]	?	Very low (not assessed)
PQoL	Nikan, 2016	Only 8 week assessment performed [6]	?	Very low (not assessed)
SF-36	Jansen, 2007<br><br>Lagaert, 2017<br><br>Hoedjes, 2011<br><br>Bahrami, 2014<br><br>Trivino-Juarez, 2017	All domains of SF-36 except General Health significantly increased between week 1 and 6)[18]<br><br>No change between week 6 and 6 months[40]<br><br>Improvement between 6-12 weeks in pre-eclamptic women[27]<br><br>Improvement in scores between 6-8 weeks and 12-14 weeks[41]<br><br>No change between 6 weeks and 6 months in >75% subgroups for all delivery modes except forceps delivery[42]	+	Low (2 studies with adequate response rates, 2 with low response rates, 1 response rate not stated, missingness reported in 1 study inconsistency among study findings)

© 2021 Sultan P et al. *JAMA Network Open*.

SF-12	Hiscock, 2007<br><br>Nolens, 2018<br><br>Norhayati, 2016<br><br>Bechard, 2019	Similar scores at 10 and 12 months but no statistical analysis[43]<br><br>Increased scores between 6 weeks and 6 months but no statistical analysis [33]<br><br>Increased scores between 1 and 6 months but no statistical analysis[34]<br><br>Not formally assessed. Physical qualitatively improves whereas mental domain decreases from month 3 to 12[35]	?	Very low (low response rates, no missingness data reported)
EQ5D	Turkstra, 2017<br><br>Turkstra, 2013<br><br>Nieminen, 2016<br><br>Kohler, 2018<br><br>Jansen, 2007	No change in total score between 2nd trimester and 6 weeks[44] (1 dimension improved)<br><br>No statistics performed. Qualitative increase from 6 weeks to 6 months and decrease from 6 months to 1 year[45]<br><br>No change up to 8 weeks [46]<br><br>Improved between 0-3; 3-7 and 21-30 days[38]<br><br>Improvement over weeks 1, 3 and 6 after all delivery modes[18]	+/-	Low (although 2 RCTs, all studies downgraded due to potential for responder bias - attrition / selection bias; 3 studies with low response rates, 1 with good response rate, 1 not reporting response rate; missingness reported in 1 study; number of eligible women not reported in 1 study)
WHOQoL-BREF		Not assessed	?	Very low (not assessed)
Nottingham Health Profile	Baghirzada, 2013	Improved scores from 1-2 days to 1-2 weeks in 5/6 domains following SVD and 4/6 following CD, but no statistical analysis [21]	?	Very low (missingness not reported; no statistical analysis; not formally assessed)
WHO disability assessment scale-II	Senturk, 2012	Not assessed (2 Postpartum time points not assessed) but scores improved from pregnancy to 8 weeks postpartum)[23]	?	Very low (not formally assessed)
QoL inventory	Nieminen, 2016	No change in score from week 1 to week 8[46]	-	Low (downgraded due to low numbers; no missingness data)
Swedish HRQoL		Not assessed	?	Very low (not assessed)

IFSAC=Inventory of Functional Status After Childbirth; Barkin=Barkin Index of Maternal Functioning; RPQoL=Rural postpartum Quality of Life; PQoL=Postpartum Quality of Life; SF-36=36-item Short Form Health Survey; SF-12=12-item Short Form Health Survey; EQ5D=European Quality of Life-5 Dimensions questionnaire; WHOQOL-BREF=World Health Organization Quality of Life-BREF score; Nottingham HP=Nottingham Health Profile; WHO DAS-II =World Health Organization disability assessment scale- II); QoL=Quality of Life; HRQoL=Health related quality of life; EPDS=Edinburgh Postnatal Depression Scale; PSC=Postpartum Symptom Checklist; PPD=postpartum depression
CFA= confirmatory factor analysis; RMSEA=root mean square error of approximation; DIF=differential item functioning; ICC=intraclass correlation coefficient; SVD=spontaneous vaginal delivery; CD=cesarean delivery; PPD=postpartum depression; ITU=intensive care; RCTs=randomized controlled trials
Overall rating presented as: Sufficient (+), Insufficient (-), Inconsistent (+/-) or Indeterminate (?); GRADE quality of evidence presented as **(**High, Moderate, Low or Very Low)

© 2021 Sultan P et al. *JAMA Network Open*.

eReferences.

1 Noor N, Aziz A, Mostapa M, *et al.* Validation of the Malay Version of the Inventory of Functional Status after Childbirth Questionnaire. *Biomed Res Int* 2015;**2015**:972728.
2 Barkin J, Wisner K, Wisniewski S. The Psychometric Properties of the Barkin Index of Maternal Functioning. *J Obs Gynecol Neonatal Nurs* 2014;**43**:792–802.
3 Hill P, Aldag J, Hekel B, *et al.* Maternal Postpartum Quality of Life Questionnaire. *J Nurs Meas* 2006;**14**:205–20.
4 Sheil E, Bull M, Moxon B, *et al.* Concerns of Childbearing Women: A Maternal Concerns Questionnaire as an Assessment Tool. *J Obstet Gynecol Neonatal Nurs* 1995;**24**:149–55.
5 Huang K, Tao F, Liu L, *et al.* Does delivery mode affect women's postpartum quality of life in rural China? *Midwifery Perinat Care* 2012;**21**:1534–43.
6 Nikan F, Jafarabadi M, Mohammad-Alizadeh-Charandabi S, *et al.* Psychometric Properties of the Iranian Version of a Postpartum Women's Quality of Life Questionnaire (PQOL): A Methodological Study. *Iran Red Crescent Med J* 2016;**18**:e35460.
7 McVeigh C. Investigating the ralationship between satisfaction with social support and functional status after childbirth. *Am J Matern Child Nurs* 2000;**25**:25–30.
8 Aktan N. Functional status after childbirth and related concepts. *Clin Nurs Res* 2010;**19**:165–80.
9 Mirghafourvand M, Mohammad-Alizadeh-Charandabi, S, Jafarabadi M, Soltanpour S. Psychometric Properties of the Iranian Version of the Inventory of Functional Status after Childbirth (IFSAC). *Iran Red Crescent Med J* 2016;**19**:e30210.
10 McVeigh C, Chaboyer W. Reliability and validity of the Inventory of Functional Status after Childbirth when used in an Australian population. *Nurs Heal Sci* 2002;**4**:107–12.
11 Fawcett J, Tulman L, Myers S. Development of the inventory of functional status after childbirth. *J Nurse Midwifery* 1988;**33**:252–60.
12 Barkin J, Wisner K, Bromberger J, *et al.* Development of the Barkin Index of Maternal Functioning. *J Women's Heal* 2010;**19**:2239–46.
13 Barkin J, McKeever, Lian B, *et al.* Correlates of Postpartum Maternal Functioning in a Low-Income Obstetric Population. *J Am Psychiatr Nurses Assoc* 2017;**23**:149–58.
14 Chamgurdani F, Barkin J, Curry C, *et al.* Comparison of Maternal Functioning between Iranian Mothers with and without Depressive Symptoms: A Case-Control Study. *Int J Env Res Public Heal* 2020;**17**:3350.
15 Shamsabi S, Barkin J, Ghanbari-Homayi S, *et al.* The Relationship between Maternal Functioning andMental Health after Childbirth in Iranian Women. *Int J Env Res Public Heal Res Publ* 2020;**17**:1558.
16 Maloni J, Park S. Postpartum Symptoms After Antepartum Bed Rest. *J Obstet Gynecol Neonatal Nurs* 2005;**34**:163–71.
17 Fishbein E, Burggraf E. Early postpartum discharge: how are mothers managing? *J Obs Gynecol Neonatal Nurs* 1998;**27**:142–8. doi:https://doi.org/10.1111/j.1552-6909.1998.tb02604.x
18 Gerard Jansen A, Essink-Bot M, Duvekot J, *et al.* Psychometric evaluation of health-related quality of life measures in women after different types of delivery. *J Psychosom Res* 2007;**63**:275–81. doi:10.1016/j.jpsychores.2007.06.003.
19 Jansen A, Duvekot J, Hop W, *et al.* New insights into fatigue and health-related quality of life after delivery. *Acta Obstet Gynecol* 2007;**86**:579–84.
20 Webster J, Nicholas C, Velacott C, *et al.* Validation of the WHOQOL-BREF among

© 2021 Sultan P et al. *JAMA Network Open*.

women following childbirth. *Aust New Zeal J Obstet Gynaecol* 2010;**50**:132–7.
21 Baghirzada L, Downey K, Macarthur A. Assessment of quality of life indicators in the postpartum period. *Int J Obstet Anesth* 2013;**22**:209–16.
22 Barkin J, Willis G, Hawkins K, *et al.* Semantic Assessment of the Barkin Index of Maternal Functioning in a Medically Underserved Obstetric Population. *Perspect Psychiatr Care* 2017;**53**:95–103.
23 Senturk V, Hanlon C, Medhin G, *et al.* Impact of perinatal somatic and common mental disorder symptoms on functioning in Ethiopian women: The P-MaMiE population-based cohort study. *J Affect Disord* 2012;**136**:340–9.
24 Hill P, Aldag J. Maternal Perceived Quality of Life Following Childbirth. *JOGNN J Obstet Gynecol Neonatal Nurs* 2007;**36**:328–34.
25 Norhayati M, Hazlina N, Aniza A. Functional status of women with and without severe maternal morbidity: A prospective cohort study. *Women and Birth* 2016;**2016**:443–9.
26 Lydon-Rochelle M, Holt V, Martin D. Delivery method and self-reported postpartum general health status among primiparous women. *Paediatr Perinat Epidemiol* 2001;**15**:232–40.
27 Hoedjes M, Berks D, Vogel I, *et al.* Poor Health-related Quality of Life After Severe Preeclampsia. *Birth* 2011;**38**:246–55.
28 Sadat Z, Abedzadeh-Kalahroudi A, Atrian M, *et al.* The impact of postpartum depression on quality of life in women after child’s birth. *Iran Red Crescent Med J* 2014;**16**:e14995.
29 Torkan B, Parsay S, Lamyian M, *et al.* Postnatal quality of life in women after normal vaginal delivery and caesarean section. *BMC Pregnancy Childbirth* 2009;**9**:1–7.
30 Handa V, Zyczynski H, Burgio K, *et al.* The impact of fecal and urinary incontinence on quality of life 6 months after childbirth. *Am J Obs Gynecol* 2007;**197**:636.e6.
31 Lin Y, Chang S, Hsieh W, *et al.* Persistent stress urinary incontinence during pregnancy and one year after delivery; its prevalence, risk factors and impact on quality of life in Taiwanese women: An observational cohort study. *Taiwan J Obstet Gynecol* 2018;**57**:340–5.
32 MacArthur C, Wilson D, Herbison P, *et al.* Urinary incontinence persisting after childbirth: extent, delivery history, and effects in a 12–year longitudinal cohort study. *BJOG An Int J Obstet Gynaecol* 2016;**123**:1022–9.
33 Nolens B, van den Akker T, Lule J, *et al.* Birthing experience and quality of life after vacuum delivery and second-stage caesarean section: a prospective cohort study in Uganda. *Trop Med Int Heal* 2018;**23**:914–22.
34 Norhayati M, Hazlina N, Aniza A. Immediate and long-term relationship between severe maternal morbidity and health-related quality of life: a prospective double cohort comparison study. *BMC Public Health* 2016;**16**:1–11.
35 Bechard F, Castelli C, Alonso S, *et al.* Impact of mode of delivery of twins on the pelvic floor 3 and 12 monthspost-partum—part II. *Int Urogynecol J* 2019;**30**:893–9.
36 Petrou S, Kim S, McParland P, *et al.* Mode of Delivery and Long-Term Health-Related Quality-of-Life Outcomes: AProspective Population-Based Study. *Birth* 2017;**44**:110–9.
37 Seppanen P, Sund R, Ala-Kokko T, *et al.* Obstetric patients’ health-related quality of life before and afterintensive care. *Aust Crit Care* 2019;**32**:116–21.
38 Kohler S, Annerstedt K, Diwan V, *et al.* Open Access Postpartum quality of life in Indian women after vaginal birth and cesarean section: a pilot study using the EQ-5D-5L descriptive system. *BMC Pregnancy Childbirth* 2018;**18**:1–13.

© 2021 Sultan P et al. *JAMA Network Open*.

39 Carlander A, Andolf E, Edman G, *et al.* Health-related quality of life five years after birth of the first child. *Sex Reprod Healthc* 2015;**6**:101–7.
40 Lagaert L, Weyers S, van Kerrebroeck H, *et al.* Postpartum dyspareunia and sexual functioning: aprospective cohort study. *Eur J Contracept Reprod Heal Care* 2017;**22**:200–6.
41 Bahrami N, Karimian Z, Bahrami S, *et al.* Comparing the postpartum quality of life between six to eight weeks and twelve to fourteen weeks after delivery in Iran. *Iran Red Crescent Med J* 2014;**16**:e16985.
42 Trivino-Juarez J, Romero-Ayuso D, Nieto-Pereda B, *et al.* Health related quality of life of women at the sixth week and sixthmonth postpartum by mode of birth. *Women and Birth* 2017;**30**:29–39.
43 Hiscock H, Bayer J, Gold L, *et al.* Improving infant sleep and maternal mental health: a cluster randomised trial. *Arch Dis Child* 2007;**92**:952–8.
44 Turkstra E, Mihala G, Scuffham P, *et al.* An economic evaluation alongside a randomised controlled trial onpsycho-education counselling intervention offered by midwives toaddress women's fear of childbirth in Australia. *Sex Reprod Healthc* 2017;**2017**:1–6.
45 Turkstra E, Gamble J, Creedy D, *et al.* PRIME: imact of previous mental health problems on health-related quality of life in women with childbirth. *Arch Womens Ment Heal* 2013;**16**:561–4.
46 Nieminen K, Berg I, Frankenstein K, *et al.* Internet-provided cognitive behaviour therapyof posttraumatic stress symptoms followingchildbirth - a randomized controlled trial. *Cogn Behav Ther* 2016;**45**:287–306.

© 2021 Sultan P et al. *JAMA Network Open*.
